# Heterogeneous agricultural robots: a review of collaborative sensing and control, challenges and opportunities

**DOI:** 10.3389/fpls.2026.1878431

**Published:** 2026-08-19

**Authors:** Rongrong Gu, Songda Luan, Yunming Zhao, Shengyi Zhao, Xi Kan, Yuxuan Ma, Tao Teng, Yuxin Sun, Chengyuan Li, Xudong Sun, Jizhang Wang

**Affiliations:** 1 School of IoT Engineering, Wuxi University, Wuxi, China; 2 Jiangsu Provincial Key Laboratory of Vehicle-Road Multimodal Perception and Control, Wuxi University, Wuxi, China; 3 College of Control Science and Engineering, Zhejiang University, Hangzhou, China; 4 School of Agricultural Engineering, Jiangsu University, Zhenjiang, China; 5 College of Mechanical and Vehicle Engineering, Chongqing University, Chongqing, China; 6 China Society of Automotive Engineers, Beijing, China

**Keywords:** air–ground–underground integration, collaborative sensing and control, heterogeneous agricultural robots, intelligent precision agriculture, multi-agent systems

## Abstract

A critical shift towards improved efficiency is currently taking place in modern agriculture, sustainability, and intelligence, driven by population growth, resource constraints, labor shortages, and increasing environmental pressure. Agricultural robots, especially heterogeneous robotic systems, have emerged as key enablers of this transformation by enabling precise, automated, and large-scale operations. This paper presents a comprehensive review of collaborative sensing and control technologies for heterogeneous agricultural robots working in both structured agricultural environments (greenhouses, orchards, and facility cultivation) and representative field farming scenarios. Focusing on recent advances over the past three years, we systematically analyze five representative robotic platforms: sprinkler irrigation robots, unmanned ground vehicles (UGVs), unmanned aerial vehicles (UAVs), agricultural robotic arms, and underground robots, from the perspectives of sensing, control, and cooperative operation. The review highlights key control methodologies, including intelligent path planning, adaptive and learning-based control, visual navigation, multi-sensor fusion, and energy-aware optimization. Furthermore, air–ground–underground integrated systems are discussed as an emerging paradigm for full-space agricultural coverage. Beyond technological progress, this paper identifies critical challenges in heterogeneous agricultural robot systems, including cooperative communication reliability, real-time safe control under dynamic environments, energy and endurance constraints, and system-level security. Finally, future research directions are outlined, emphasizing integrated communication and sensing, dual-arm and multi-arm collaborative manipulation, and heterogeneous multi-robot cooperative control frameworks.

## 1 Introduction

Currently, global agriculture is undergoing a critical transition from traditional, extensive production models to modern, precise, and intelligent operational paradigms. This transformation is driven by both external systemic pressures and internal structural constraints: externally, the intertwined challenges of population growth, finite natural resources, and escalating environmental stress have created increasingly prominent contradictions in food supply security and ecological sustainability; internally, the persistent shortage of agricultural labor and the deepening trend of population aging have become core bottlenecks restricting the long-term sustainable development of the agricultural sector. Against this backdrop, intelligent agricultural robotic technology has emerged as a core technical pathway to address these challenges, among which high-precision control systems and reliable communication architectures serve as fundamental enablers for efficient, stable, and collaborative robotic operations in complex agricultural environments.

Quantitative data from global agricultural research institutions further illustrate the urgency of this transformation. According to a 2024 report from the Food and Agriculture Organization of the United Nations (FAO) (FAO et al., 2024), the global population is projected to exceed 9.7 billion by 2050, requiring a 60% increase in food supply relative to current levels to maintain global food security. However, global arable land resources remain strictly limited: soil degradation, salinization, and continuous urban expansion have led to a sustained decline in the area of high-quality arable land year on year. Compounding these resource constraints, traditional extensive agricultural production is associated with low water use efficiency, widespread non-point source pollution from excessive fertilizer and pesticide application, and growing vulnerability to extreme climate events such as droughts and floods, all of which have imposed substantial resource and environmental burdens on agricultural development. In parallel, the global agricultural labor force is facing structural contraction and aging: with accelerating urbanization, the total number of agricultural workers worldwide has shown a sustained downward trend, and the aging of the labor force has become a universal phenomenon. For instance, the average age of agricultural workers in China has reached approximately 50 years, with a severe shortage of laborers under 35 years of age (Li et al., 2024; Tong et al., 2024); while developed countries in Europe and North America have partially offset labor shortages through mechanization, rising agricultural labor costs still account for a large share of total production costs, and the dual pressure of labor supply gaps and cost increases has significantly constrained improvements in agricultural production efficiency.

To mitigate crop yield losses induced by extreme climate events and overcome the limitations of traditional experience-dependent production modes, modern agricultural development urgently requires a systematic transition toward high-stability, high-efficiency, and highly automated production through technological innovation. Intelligent heterogeneous agricultural robotic systems, which integrate multiple complementary operational platforms, have been widely recognized as a core technical solution for next-generation precision agriculture. Within such systems, control technology directly determines the accuracy, stability, and operational safety of individual robots and multi-robot clusters, covering core performance indicators such as trajectory tracking precision, task execution accuracy, and dynamic collision avoidance capability. Communication technology, meanwhile, acts as the fundamental backbone for collaborative operation of heterogeneous robotic systems, supporting real-time data interaction, distributed task scheduling, and dynamic state synchronization across different platforms. The overall performance of control and communication systems directly restricts the operational efficiency, deployment cost, and application scope of agricultural robotic systems in structured agricultural scenarios such as commercial greenhouses and orchards, and has thus become a core direction of current technological research and iterative upgrading in this field.

**Figure 1:**
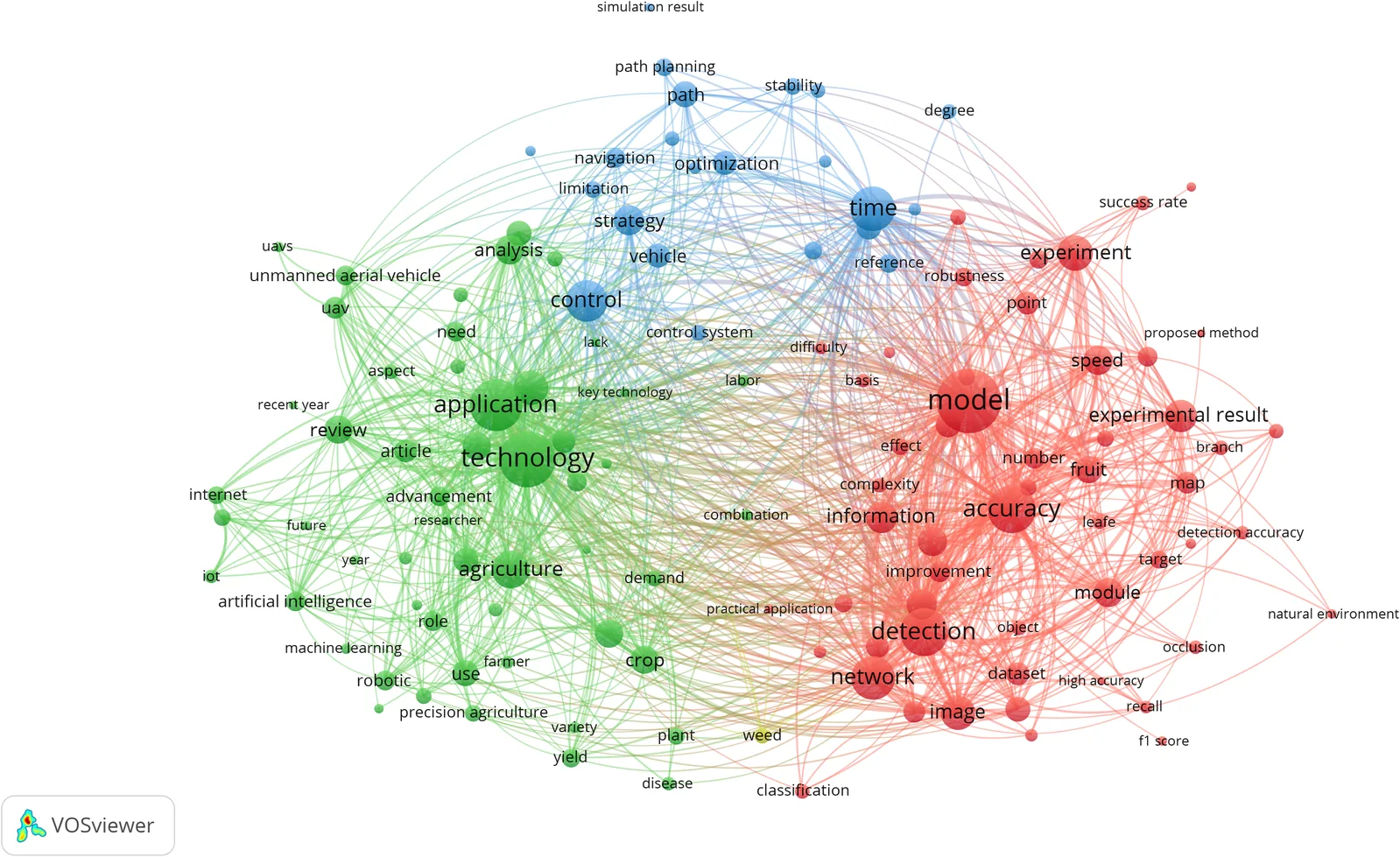
Keyword co-occurrence network on agricultural intelligent robot control.

To systematically sort out the research hotspots, evolutionary context, and internal correlations in the field of agricultural intelligent robot control, this study constructs a keyword co-occurrence network of relevant research, as shown in **Figure 1**. The keyword co-occurrence analysis was conducted using VOSviewer version 1.6.20. The bibliographic records used for this analysis were retrieved from Web of Science Core Collection on March, 2026. The complete search query was: “agricultural AND robot control” A total of 347 records published between March 2023 and March 2026 were included in the bibliometric analysis. The analysis was based on all keywords, including author keywords and index keywords, using the full counting method. Before analysis, synonymous terms, abbreviations, singular and plural forms, and spelling variants were manually standardized using a thesaurus file. The minimum occurrence threshold was set at 15 occurrences. Of the 11439 extracted keywords, 83 met the threshold and were included in the final network. The network was normalized using the association-strength method, and the default VOSviewer clustering algorithm. It should be noted that this analysis was intended to provide an overview of the general research hotspots and thematic relationships within the retrieved literature, rather than to characterize only the studies finally included in the systematic qualitative synthesis. In this network, the color of nodes differentiates research maturity: red marks high-density, well-studied research areas, green represents rapidly developing emerging fields, and blue indicates relatively underexplored research directions. The size of each circle corresponds to the occurrence frequency of the corresponding keyword, while the connecting lines between nodes reflect the strength of academic correlation between different research topics.

This review focuses on control technologies and collaborative mechanisms of heterogeneous agricultural robots and robot clusters, with closed structured agricultural environments (commercial greenhouses, orchards, and facility cultivation) as the core research scenario, and also includes representative field-scale heterogeneous robotic systems for comparative analysis and technical reference.

To ensure the timeliness and systematic rigor of the review, literature published in the past three years is selected as the core analysis object, and the literature screening process strictly follows the PRISMA 2020 systematic review specification. Specifically, a comprehensive literature search was conducted across four mainstream academic databases: Web of Science Core Collection, Scopus, IEEE Xplore, and ScienceDirect. The search was executed on March 20, 2026, covering studies published between January 2023 and March 2026.

The complete search strategy combined keywords related to heterogeneous agricultural robots, collaborative sensing, cooperative control, and structured agricultural scenarios. The core retrieval equation adapted for each database is as follows:


TS=((“heterogeneous agricultural robot*” OR “agricultural multi−robot system*”) AND (“collaborativesensing” OR “cooperative control” OR “multi−agent coordination”) AND (“greenhouse” OR “orchard”OR “structured agriculture” OR “facility agriculture”))

**Figure 2:**
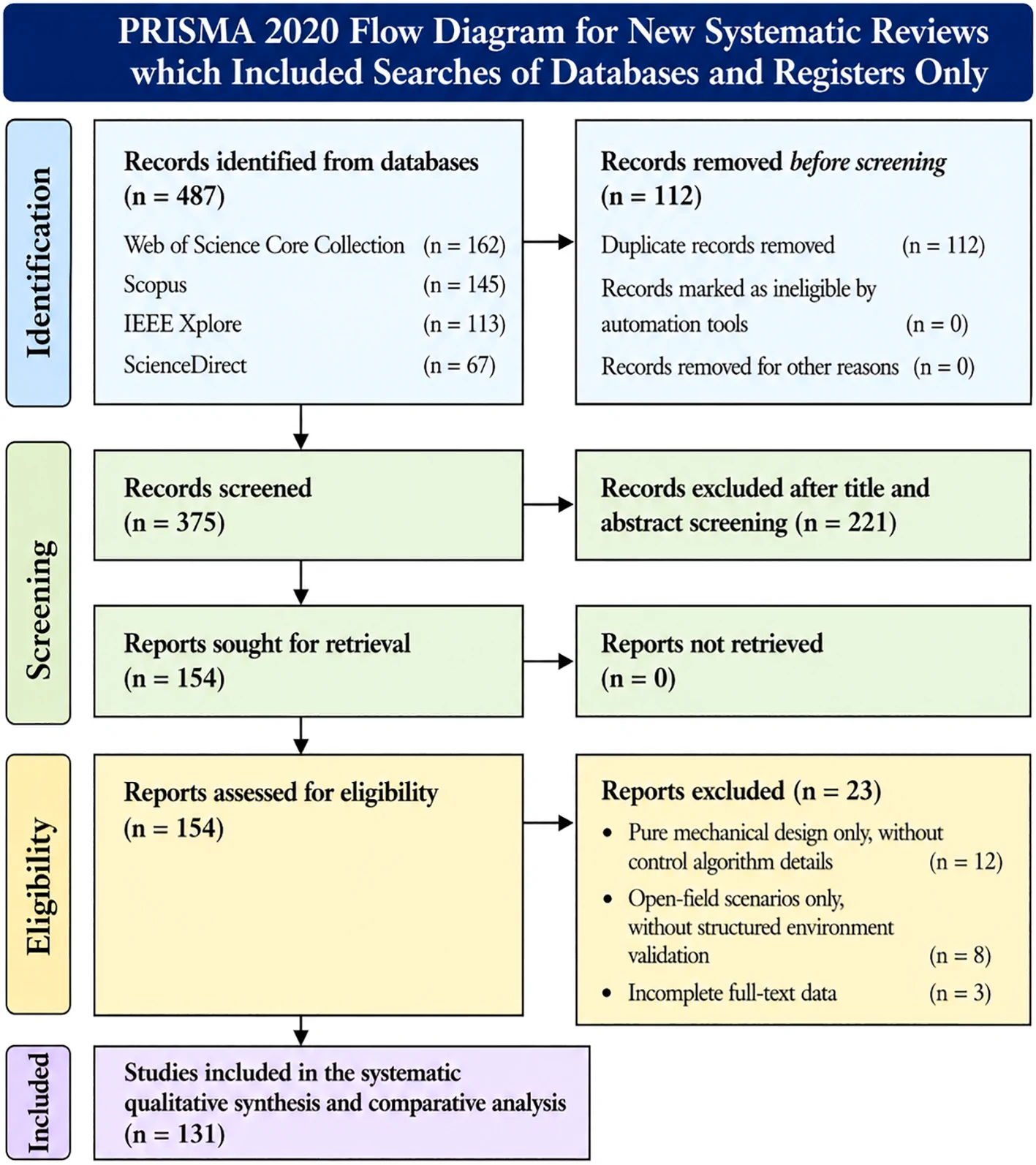
PRISMA 2020 flow diagram showing the identification, screening, eligibility assessment, and inclusion of studies in this systematic review.

A total of 487 records were initially retrieved: 162 from Web of Science Core Collection, 145 from Scopus, 113 from IEEE Xplore, and 67 from ScienceDirect. After removing 112 duplicate records, 375 records entered the title and abstract screening stage. During this stage, 221 records out of the research scope were excluded, leaving 154 records for full-text eligibility assessment. After full-text review, 23 records were further excluded: 12 focused on pure mechanical design without control algorithm details, 8 only involved open-field scenarios without structured environment validation, and 3 had incomplete full-text data. Finally, a total of 131 studies meeting the predefined inclusion criteria were included for systematic qualitative synthesis and comparative analysis. As shown in **Figure 2**, a total of 487 records were identified, of which 131 studies were finally included in the systematic qualitative synthesis.

Existing review studies in the field of agricultural robotics have provided valuable summaries of technological progress in related domains, but notable research gaps remain. Most existing reviews focus on the overall development status of agricultural robotic technology or technical advances of single-type robotic platforms, with relatively limited systematic discussion specifically targeting heterogeneous multi-robot systems. In terms of technical dimensions, many reviews emphasize hardware system design, sensor configuration, and application scenario expansion, while lacking in-depth sorting and comparative analysis of core control algorithms and communication coordination mechanisms, which are the core enablers of multi-robot collaborative operation. In addition, most existing reviews adopt a broad agricultural scenario scope, and few studies focus specifically on closed structured environments such as greenhouses and orchards, resulting in insufficient pertinence of the summarized technical frameworks and control schemes for specific application scenarios. Furthermore, given the rapid iteration of related technologies in recent years, timely systematic reviews covering the latest research advances over the past three years remain relatively scarce.

Against this background, this review aims to systematically sort out the latest research advances in heterogeneous agricultural robotic systems in closed structured agricultural scenarios over the past three years, with a core focus on control technologies and communication coordination mechanisms. The main contributions of this work are summarized as follows:

1. This paper provides a comprehensive, control-oriented systematic review of recent research on heterogeneous agricultural robots in both structured agricultural environments (greenhouses, orchards) and typical field scenarios. Focusing on studies published in the past three years, this review systematically covers control-related technological advances across five categories of robotic platforms: unmanned aerial vehicles, unmanned ground vehicles, sprinkler irrigation robots, agricultural robotic manipulators, and underground operating robots, forming a unified classification framework of air-ground-underground heterogeneous systems
2. This review summarizes and compares representative control methodologies for different types of agricultural robotic platforms, covering core technical directions including path planning and trajectory tracking, formation and cooperative control, adaptive and learning-based control, and communication- assisted control frameworks. Particular attention is paid to air-ground-underground integrated collaborative systems, elaborating on how multi-robot coordination and control mechanisms support efficient, safe, and precise agricultural operations.
3. From the perspective of control and communication collaboration, this paper identifies the key technical challenges currently faced by heterogeneous agricultural robotic systems, and summarizes prospective future research directions corresponding to these challenges, including robust cooperative control under communication uncertainty, real-time safety assurance and collision avoidance in dynamic environments, energy-aware multi-robot scheduling and control, and scalable heterogeneous multi-agent coordination mechanisms, providing reference insights for the development of reliable and intelligent agricultural robotic systems.

Several review studies have investigated agricultural robotics from different perspectives, including robotic platforms, sensing technologies, automation systems, and agricultural applications. However, most existing reviews mainly focus on specific robotic platforms or individual application scenarios, while the integration of heterogeneous robotic systems, collaborative sensing, communication architectures, and cooperative control strategies has received limited attention. To further clarify the differences between existing reviews and this work, a comparison of representative agricultural robotics reviews is summarized in **Table 1**.

**Table 1:** Methodological and thematic comparison between the present systematic review and existing reviews on agricultural robotics.

Ref	Review type	Date range	Review approach	Main scope and platforms	Control and coordination	Specific gap addressed by the present review
(Jin and Han, 2024)	Comprehensive review	2018–2023	Narrative	Ground-based robotic arms; hardware and software; applications in greenhouses, fields, and orchards	Perception, motion planning, and manipulator control; primarily focused on individual robotic-arm platforms	Does not systematically compare multi-robot coordination, heterogeneous collaboration, or validation maturity
(Barbosa Junior et al., 2024)	Systematic state-of-the-art review	1988–2024	Systematic, PRISMA-guided	Ground robots for specialty crops; application areas, benefits, limitations, and bibliometric trends	Application-oriented analysis with limited algorithm-level comparison of control and inter-robot coordination	Requires a control-centred synthesis covering single-platform, homogeneous, and heterogeneous robotic systems
(Droukas et al., 2023)	Survey review	NR	Narrative	Ground agricultural and harvesting robots; vision, navigation, human–robot interaction, grasping, and end-effectors	Component- and task-level control, mainly for a mobile platform equipped with one manipulator	Does not distinguish robot composition, validation type, operating environment, or collaborative architecture
(Thaker, 2023)	State-of-the-art review	NR	Narrative	Broad agricultural operations; artificial intelligence, sensing, autonomous systems, and cooperative robots	Control and cooperation are discussed descriptively rather than comparatively	Lacks a reproducible review protocol and an evidence-based comparison of control and coordination methods
(Singh et al., 2024)	Conference review	NR	Narrative	Agricultural robotics applications, AI/ML, IoT, autonomous vehicles, challenges, and future prospects	High-level discussion of automation and robot operation, with limited treatment of multi-robot coordination	Does not provide a validation taxonomy or a systematic synthesis of heterogeneous multi-robot operation
(Spagnuolo et al., 2025)	Technical review and market survey	2023–2024	Narrative technical survey	Commercial and prototype agricultural robots for field and greenhouse operations; functions, crops, power systems, and locomotion	Focuses mainly on product and system characteristics rather than control algorithms	Does not compare control algorithms, collaboration mechanisms, communication architectures, or experimental evidence
(Nkwocha et al., 2025)	Comprehensive technology review	2015–2025	Systematic search, non-PRISMA reporting	UAVs, UGVs, USVs, and robotic arms; sensing, control, networking, swarm robotics, and coordination	Broad coverage of control and networking, but limited classification by robot composition and validation environment	Requires deeper comparison of single-platform, homogeneous, and heterogeneous systems under different validation conditions
This review	Systematic qualitative review	Jan. 2023–Mar. 2026	Systematic, PRISMA 2020	Single-platform autonomy and homogeneous or heterogeneous multi-robot operation in fields, greenhouses, orchards, and structured agricultural environments	Comparative analysis of control methods, coordination mechanisms, communication architectures, validation types, and operating environments	Integrates control technology, collaborative mechanisms, robot composition, environmental conditions, and deployment maturity within a unified framework

NR indicates that the literature coverage period was not reported. The classification as systematic or narrative was based on whether the review reported a reproducible database search, eligibility criteria, and an explicit screening procedure.

It should be clarified that the literature cited in this review is divided into three categories:

1. Core included studies for the systematic review: A total of 131 relevant references are cited in this article. Among them, 117 studies published from January 2023 to March 2026 serve as the primary subjects for qualitative synthesis and comparative analysis in this work, and the core analysis of this review is conducted based on literature published within this period.
2. Foundational background literature: A total of 14 early studies published before 2023 are cited in this paper. They are only used for background description and basic concept elaboration and are not incorporated into the analytical framework of the systematic review.
3. Published review articles for comparison: Existing review papers adopted for horizontal comparative analysis and identification of research gaps, which do not belong to the core analysis dataset.

All core included studies strictly follow the time range and inclusion criteria, while foundational literature and comparative reviews are clearly distinguished in narrative and table annotations.

## 2 Overview of agricultural robot swarms

Before elaborating on specific platforms, a unified classification system for agricultural robotic systems is first defined to clarify conceptual boundaries:

- Single-robot autonomous system: A single robotic platform with independent perception, decision-making and execution capabilities, without multi-robot cooperative interaction.
- Homogeneous multi-robot system: A system composed of multiple robots with identical mechanical structure, functional attributes and motion modes, such as UAV swarms and UGV fleets.
- Heterogeneous multi-robot system: A collaborative system composed of robots with different structures, motion modes and functional capabilities, such as air-ground cooperative systems composed of UAVs and UGVs.
- Mobile manipulator system: An integrated platform combining a mobile ground base and a robotic manipulator, with both mobility and precision operation capabilities.
- Distributed agricultural sensor network: A networked perception system composed of spatially dispersed sensor nodes, providing environmental data support for robotic systems.
- Air-ground-underground integrated system: A typical heterogeneous multi-robot system covering three spatial dimensions, integrating aerial UAVs, ground platforms and underground sensing/operation robots.

In the following analysis and tables, each cited study is marked with its corresponding system category to clarify the scope of technical discussion.

Agricultural robot swarms are progressively evolving into heterogeneous cyber–physical systems operating across airborne, ground, and belowground domains. Although these platforms differ in mechanical configuration, mobility constraints, and deployment environments, their development is fundamentally driven by shared control-theoretic challenges. These include constrained trajectory optimization, safety-critical tracking under uncertainty, adaptive environmental regulation, nonlinear stabilization of complex dynamics, perception–control coupling, and networked coordination under communication and energy constraints.

From a control-oriented perspective, agricultural robotic systems can no longer be treated as isolated hardware units. Instead, they form spatially distributed and functionally interconnected layers within an integrated intelligent agricultural framework. Accordingly, this section reorganizes representative agricultural robots according to spatial deployment (air, ground, and underground) while emphasizing their underlying control paradigms and system-level coordination mechanisms.

**Figure 3:**
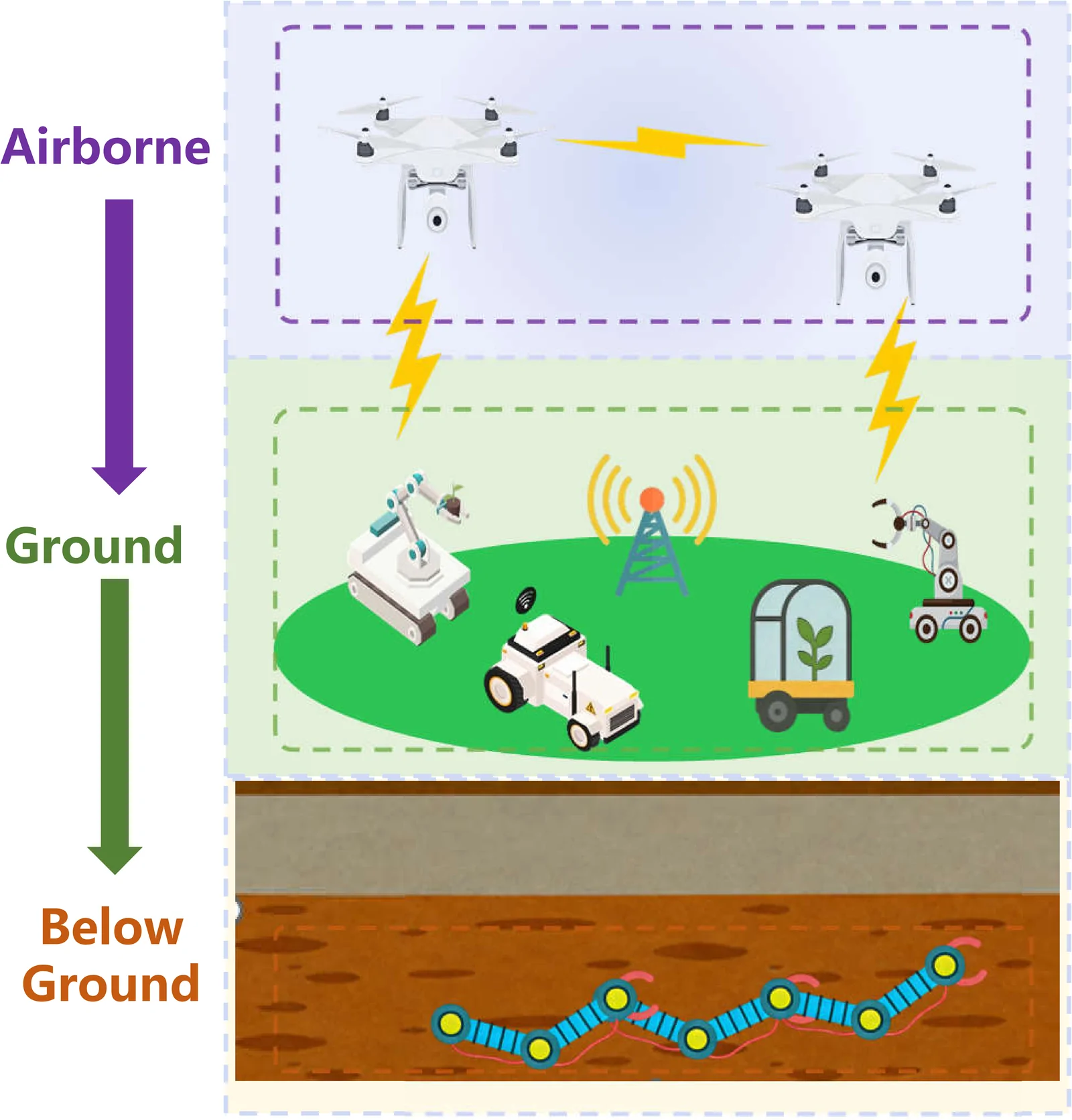
Airborne, ground, and belowground agricultural robots.

**Figure 3** illustrates the architecture of the integrated air ground underground agricultural robotic system. The left part depicts the air–ground linkage network, where UAVs provide large-scale perception and supervisory planning for ground platforms. The right part shows the interaction between ground and underground systems, enabling *in-situ* sensing and soil-level intervention. Together, these layers construct a full-space operational architecture covering aerial monitoring, ground-level execution, and subsurface sensing. Such integrated systems significantly enhance the scalability, precision, and automation potential of intelligent facility agriculture.

**Figure 4:**
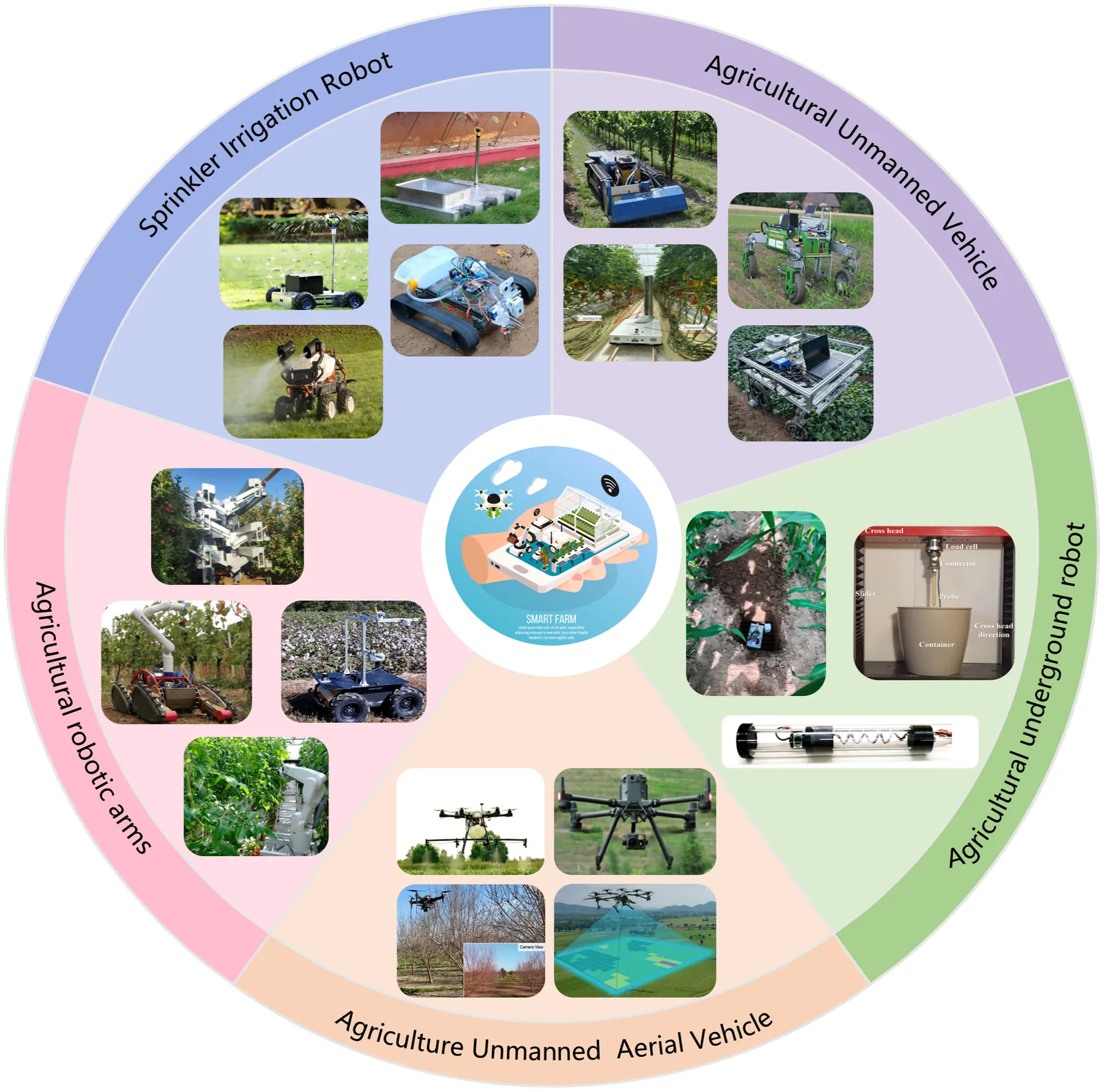
Classification of agricultural intelligent robots.

From a control-oriented perspective, this section for the first time integrates the control technologies of four core robotic platforms: sprinkler irrigation robots, unmanned ground vehicles (UGVs), unmanned aerial vehicles (UAVs), and agricultural robotic manipulators, and clarifies the correlations between their core control and sensing modules. **Figure 4** lists the common working scenarios and common design forms of the four core robots introduced in this section.

### 2.1 Airborne systems: constrained optimal planning and adaptive flight control

Due to the flexibility of flight, Drones can accomplish many tasks in agricultural production (Yumnam et al., 2025). mentions that there are still many challenges in the education of farmers in operating drones, the flight time of drones, privacy and data security.

Similar to the unmanned vehicle, the drones have revolutionary potential in control and sensing cooperation aspects. For example (Verma, 2025), synchronized of drone technology with technologies such as machine learning and computer vision.

In the control aspect (Palta et al., 2025), proposed the unmanned aerial vehicle (UAV) path planning and navigation based on Dijkstra (Wei et al., 2025). also takes into account the characteristics of the agricultural closed environment and proposes a navigation method for unmanned aerial vehicles The operations of unmanned aircraft usually have the feature of multiple flights. Aiming at this issue (Dong et al., 2025), propose a control method based on comprehensive coverage path planning and an improved A* algorithm. According to the summary in (Merei et al., 2025), it has outlined the typical algorithmic techniques for obstacle detection and avoidance in unmanned aerial vehicles over the past decade. The system establishes a holistic objective function composed of three components: coverage task cost, flight safety cost, and path length cost. The improved black-winged kite algorithm (DWBKA) is introduced to enhance the global search and obstacle avoidance capabilities in (Li et al., 2025; Merei et al., 2025). In (Amertet et al., 2024; Siwek et al., 2024), the proportional-integral-derivative (PID) algorithm has been effectively improved, which aims to dynamically adjust the PID parameters through fuzzy logic to cope with the nonlinear dynamics, parameter disturbances, and noise interference faced by the unmanned aerial vehicle in complex agricultural environments.

In the drone spraying operation, the main problem of precise spraying drones is the high cost. To reduce the waste of the spray operation, a control system based on the spray distribution variation coefficient was proposed in (Wang et al., 2024). The key objective of the system is to implement real-time prediction and control over the spray distribution variation coefficient (CV) with the assistance of the random forest regression algorithm. Moreover, technologies such as image processing technology, machine learning algorithms, convolutional neural network (CNN) model (Maity et al., 2024; Akhil et al., 2025; Borah et al., 2025; Jeba Kumar et al., 2025; Laura et al., 2025; Raj et al., 2025), the decision trees operation (Sitompul et al., 2024), and the remote sensing approach (Guan et al., 2025).

However, in greenhouse agricultural, there are still limitations and challenges in the technical aspects of precise agricultural applications in greenhouse environments. The issues are described in (Ben Miled et al., 2023; Srinil and Thongnim, 2024; Kar and Dhal, 2025), where the unmanned aerial vehicle, by carrying hyperspectral imaging (HSI) technology in an “overhead-detailed-dynamic” monitoring mode, provides multi-scale data support from “leaves to fields” for greenhouse agriculture and addresses problems such as improving accessibility in greenhouse environment and control system stability issues, and communication and data transmission bottlenecks.

From a control perspective, Dijkstra and improved A* algorithms are the prevailing approaches in global path planning for agricultural UAVs, with the former prioritizing optimality and the latter balancing efficiency and adaptability. Fuzzy PID is the most widely used flight control scheme, which effectively suppresses wind disturbance and load fluctuation at the cost of relying on expert experience for parameter tuning. The core trade-off lies in the contradiction between operation accuracy and endurance: high-frequency pose correction improves spraying uniformity but significantly reduces flight time.

### 2.2 Ground systems: safety-critical tracking and precision interaction control

Ground systems form the core execution layer of agricultural heterogeneous robots and can be further divided into two subcategories according to functional attributes: mobile ground platforms that undertake navigation and transportation tasks, and robotic manipulators that perform precision operation tasks. Sprinkler irrigation robots, as a typical special-purpose UGV, are classified under mobile ground platforms.

#### 2.2.1 Mobile ground platforms

Autonomous vehicles are increasingly being used in precision agricultural operations and are cited in (Comparetti et al., 2025). The main issue to be studied regarding the control of agricultural unmanned vehicles is the control of path tracking. (Amertet and Gebresenbet, 2025; Ghorab and Lorenzen, 2025) proposed a dynamic self-triggering intelligent path tracking control method based on quadratic programming of control barrier functions or traditional global path planner based on the Dubins Traveling Salesman Problem (DTSP) with a nonlinear model predictive control (NMPC) strategy.

Besides, another characteristic of real-time performance is avoiding potential collisions when facing special environmental conditions such as uneven terrains in traditional path tracking technologies. The improved heuristic algorithm is then applied, such as improved Sparrow Search Algorithm (Wen et al., 2025), improved grey wolf optimization algorithm (IGWO) (Zhu et al., 2025), and improved adaptive multi-modal ant colony optimization algorithm (Yongtao et al., 2025). The improved hybrid dung beetle optimization algorithm (Zhang et al., 2025) was proposed in greenhouse robot operations. These algorithms have key points including population initialization optimization, convergence factor improvement, and integration with particle swarm optimization.

With the development of computer technologies, visual navigation and AI based trajectory training also hold significant importance in the research of agricultural robots. In (Zhang et al., 2025), a path tracking control algorithm based on deep reinforcement learning was proposed. In (Xu et al., 2025), the improved Deep Deterministic Policy Gradient algorithm (IDDPG) a scheme was presented to optimize the longitudinal speed of unmanned vehicles, aiming to enhance motor efficiency and decrease energy consumption. Meanwhile, the Deep Q-Network (DQN) algorithm is employed to regulate the lateral trajectory of unmanned vehicles, so as to improve path tracking accuracy.

However, due to the limited greenhouse area with high productivity at different altitudes, greenhouse robot operations have unique features towards the path training. (Cai and Cao, 2025) proposed an improved self-supervised monocular pose tracking model based on the Monodepth2 model was proposed to deal with the Dubinas extraction of visual images is prone to problems such as crop occlusion and monotonous color and texture. (Guan et al., 2025) considered a simultaneous localization and mapping (SLAM) system based on the fusion of laser radar and inertial navigation using the A* algorithm. At the same time, the robot has an unloading mechanism control, with the functions of automatic unloading and load adaptation.

Sprinkler robots usually integrate spraying technology, sensors and perception systems, path planning and autonomous control. Through the integration of technologies, the robots designed by this method can usually achieve precise spraying, adaptation to complex environments, and many other operations.

This summary by (Lochan et al., 2024) presents the current situation of design and development, key technologies, etc. involved in sprinkler irrigation robots. Besides (Krishna et al., 2025; Peshattiwar et al., 2025), proposed monitors the soil moisture content through soil humidity sensors, which serves as the key basis for triggering irrigation. (Furqan et al., 2022; Abdelhamid et al., 2025) utilized the sustainability of energy as a research target, such as solar-powered intelligent irrigation to reduce energy consumption.

At present, more and more control conditions are applied to the operation decisions of sprinkler robots. Air temperature and soil moisture are combined as input parameters for the sprinkler robots. By simulating human decision-making logic, the sensor values are converted into fuzzy variables described in natural language, and then rule-based reasoning generates precise control instructions that meet the needs of plants. For example (Malode et al., 2025), designed an autonomous agricultural robot capable of performing tasks such as sowing and drip irrigation, while in (Subbulakshmi et al., 2025), a new sprinkler irrigation design scheme was proposed, where the field programmable gate array (FPGA) scheme allows parallel execution, thus the output will become faster in response to humidity changes.

From a control performance perspective, model-based methods represented by nonlinear model predictive control achieve the highest path tracking accuracy, but they incur high computational overhead and require on-board industrial computers for real-time solution. Improved intelligent optimization algorithms can be deployed on low-cost embedded platforms with better convergence efficiency than standard heuristic algorithms, but their stability lacks strict theoretical proof. Reinforcement learning-based schemes can reduce motor energy consumption while ensuring tracking accuracy; for sprinkler irrigation robots, the parallel control architecture based on FPGA greatly shortens the response delay of humidity-triggered irrigation.

In terms of validation level, more than half of UGV control algorithms are only verified in simulation environments, some have completed prototype tests on flat open fields, and only a small number have carried out long-term operation verification in narrow greenhouse rows or uneven orchard terrain. The core trade-off lies in the contradiction between tracking accuracy and computing resource consumption: high-precision model predictive control significantly improves operation quality, but puts forward higher requirements on on-board computing power and system cost, which is the main factor restricting its large-scale popularization in facility agriculture.

#### 2.2.2 Agricultural robotic manipulators

Lastly, agricultural robotic arms have been extensively employed in applications including greenhouses, farmlands, and orchards. In greenhouses, they can perform tasks like ground planting, table planting, and vertical planting, enhancing efficiency through visual perception and optimized end-effectors. In orchards, they are suitable for operations involving fruit trees, vines, and low-growing ground crops.

In recent studies, digital twin technology has been introduced into greenhouse robotic arm systems to build virtual models of crops and robots for pre-operation trajectory simulation and collision prediction, reducing the risk of crop damage in physical debugging.

However, the application of agricultural robotic arms still faces multi-dimensional challenges including environmental adaptability, algorithm and control complexity, as well as data backhaul constraints and security issues. For the research on trajectory tracking of the robotic arm (Liu et al., 2025), proposed a control method based on inverse kinematics and fuzzy PID algorithm. Compared with the traditional PID control, this system introduces a control strategy that uses fuzzy rules to adjust the PID parameters online in a self-adaptive manner. (Taddei Dalla Torre et al., 2025) considered a silicone material soft gripper to achieve loss-free and efficient harvest. In response to the characteristics of flexible crops, a similar harvesting task for button mushrooms was also proposed in (Xu et al., 2025). The robotic arm is equipped with a precise end effector, and the visual sensor locates the planting holes or seedbed positions, combined with the data from the soil moisture sensor for judgment. The task objective in (Khan et al., 2024; Yu et al., 2024) was to assist small-scale growers in reducing costs by harvesting tomatoes in a hydroponic system. The system is fabricated from aluminum and low-carbon steel, and is equipped with a 4-degree-of-freedom robotic arm.

Regarding the research on multi-degree-of-freedom robotic arms, the use of a two-degree-of-freedom robotic arm with visual control for autonomous target tracking was proposed in (Sahu et al., 2025). Subsequently, the concept of a six-degree-of-freedom robotic arm has been increasingly applied in agriculture. In (Qian, 2025), a design for a crop harvesting system based on image processing technology was proposed. In (Sroymuk et al., 2025), a high integration of image processing technology and six-degree-of-freedom robotic arms was proposed. The final design of the system enables the robotic arm to perform tasks in dynamic environments with minimal human intervention (Barcelos de Assis et al., 2025). also proposed a six-degree-of-freedom robotic arm control based on computer vision and machine learning, aiming to achieve the target instructions through machine vision output.

The integration of the six-degree-of-freedom robotic arm with various other technologies has great potential for agricultural operations. In (Amisha et al., 2023; Dong et al., 2024; Li et al., 2024; Purwantana et al., 2024; Subrata and Baiquni, 2024; Cheng et al., 2025; Kamei and Wang, 2025; Kulhandjian et al., 2025), a study on intelligent grasping of robotic arms based on machine vision recognition was proposed. These studies collectively focus on integrating machine vision with agricultural robotic arms to enhance perception, operation accuracy, and automation. Through visual recognition, stereoscopic imaging, deep learning, and multisensor fusion, the systems achieve crop identification, 3D localization, sorting, and precise harvesting. Various design, such as height-adjustable platforms, flexible or passive grippers, and lightweight redundant-degree-of-freedom (DOF) robotic arms, further enhance adaptability, obstacle avoidance capability, and operational efficiency in agricultural environments.

Meanwhile, in (Chang and Huang, 2024), the harvesting of strawberries was carried out with the aim of reducing fruit damage under the same hydroponic environment. The system uses a multi-joint rope-driven robotic arm with a cutting-and-clamping mechanism at the end. These articles aim to improve the control capabilities of arms.

In terms of control scheme performance, the architecture combining inverse kinematics and fuzzy PID is the mainstream control scheme for agricultural picking manipulators. Compared with traditional fixed-parameter PID, fuzzy adaptive adjustment reduces end positioning error and shortens dynamic response time. The six-degree-of-freedom manipulator combined with machine vision can achieve a high success rate of fruit grasping in static greenhouse environments; soft end-effectors made of silicone or flexible materials effectively reduce the fruit damage rate during picking, but their load capacity is generally low, and the single grasping cycle is longer than that of rigid grippers.

In terms of validation level, most agricultural manipulator control schemes are verified on laboratory bench platforms, some have completed potted crop picking tests in simulated greenhouse environments, and only a small proportion have carried out continuous operation verification in actual commercial greenhouses or orchards. The core trade-off lies in the contradiction between operation non-destructive degree and operation efficiency: flexible end-effectors reduce crop damage, but also bring about a decrease in picking speed and load capacity, which needs to be matched according to the value and physical characteristics of target crops.

### 2.3 Belowground systems: networked and energy-constrained control

Underground robots are a type of intelligent equipment specifically designed to perform specific agricultural operations within the soil or beneath the surface. With the deepening development of precision agriculture, sustainable farming, and smart farm concepts, underground robots have gradually become a research hotspot in the field of agricultural engineering due to their advantages such as non-destructive monitoring, precise intervention, and long-term perception. Currently, most systems are still in the stage of laboratory prototypes or small-scale field trials, but their technical paths have already formed a system. This article systematically reviews the main types, core functions, and control methods of current agricultural underground robots.

In (Holtorf et al., 2023), the sensor nodes were developed based on ESP32, equipped with long range radio (LoRa) communication chips and temperature/humidity sensors. The control method was achieved through a low-power protocol. Communication with UAV was conducted via the LoRa standard to realize wireless collection and transmission of underground soil parameters. Meanwhile, an autonomous sampling robot was adopted for soil sampling in (Nguyen et al., 2025). The design achieved precise control of the sampling depth and quality, and the efficiency and spatial resolution of large-scale farmland sampling were enhanced through the autonomous navigation robot.

The root system observation and imaging robot is designed to non-destructively obtain data on the structure, growth dynamics and physiological state of crop roots, serving breeding, water and fertilizer optimization, and stress response research. The groot robot is designed to complete long-term dynamic root imaging and environmental control tasks in (Rajanala et al., 2023), and is controlled by an Arduino microcontroller.

Underground pest and disease detection robots are also one of the important aspects of underground robot operations. In (Song et al., 2023), a minimally invasive root phenotyping robot MISIRoot was designed to identify root damage caused by western corn rootworm at an early stage. In (Bayrakdar, 2019), an intelligent pest detection technology for precision agriculture was proposed, which was studied through underground wireless sensor nodes and mathematical simulation models. Future research on energy consumption issues in underground sensor networks may contribute to current precision agriculture technologies.

In (Mishra et al., 2018), a soft robotic system that can transform the morphological characteristics of corn roots into mobile entities in the soil was proposed. By systematically exploring the morphological characteristics and physiological functions of plant root systems, a solid theoretical foundation and practical reference can be laid for the innovation of biomimetic technologies to address challenging environmental conditions. It is proposed in (Wu et al., 2018) that a robot platform capable of characterizing root growth enables the robot to achieve automated imaging and irrigation coordination operations. In the future, underground robots in agriculture still have great potential in developing anti-interference underground positioning technologies, improving energy density, enhancing human-machine collaboration interfaces, and promoting the construction of standardized testing platforms.

In conclusion, agricultural robot groups are the core equipment of smart agriculture. Their significance lies in breaking the traditional reliance on human labor in agriculture, significantly enhancing operational efficiency and accuracy through multi-machine collaboration, and promoting the transformation of agriculture towards unmanned and refined operations. **Table 1** summarizes the corresponding communication and sensing methods for various types of robots in this section, presenting the technical compatibility intuitively.

From the perspective of control strategy characteristics, underground agricultural systems are still dominated by threshold-triggered control and low-power duty-cycle control strategies. Underground sensor nodes based on low-power communication protocols can achieve long battery life at a low sampling frequency; autonomous soil sampling robots can realize precise control of sampling depth, with sampling efficiency significantly higher than manual sampling. For root imaging robots, the microcontroller-based closed-loop control scheme enables long-term uninterrupted dynamic observation of root systems with high repeatability of imaging position.

In terms of validation level, the vast majority of underground robotic systems are still in the stage of laboratory prototype verification or small-scale plot tests, and very few systems have completed long-term field deployment verification lasting for months. The core trade-off lies in the contradiction between sensing/operation accuracy and energy endurance: high-frequency sampling and high-precision positioning will significantly increase power consumption, while soil signal attenuation further limits communication distance and data transmission rate, which is the core bottleneck restricting the large-scale deployment of underground agricultural robots.

## 3 Challenges in heterogeneous agricultural robots

In this section, we review the key challenges of cooperative communication, sensing and control in heterogeneous agricultural robot systems. Representative studies on communication, sensing, and control across airborne, ground, robotic-arm, and below-ground agricultural robotic platforms are summarized in **Table 2**.

**Table 2:** Related work on heterogeneous networks for agricultural robots.

Category	Ref.	Communication, Sensing	Control
Airborne (Unmanned Aerial Vehicle)	(Verma, 2025)	Computer vision	–
	(Palta et al., 2025)	Dijkstra navigation	–
	(Dong et al., 2025)	Path planning	Improved A*
	(Merei et al., 2025)	DWBKA obstacle	–
	(Siwek et al., 2024)	–	Nonlinear PID
	(Wang et al., 2024)	–	Spray control
	(Kar and Dhal, 2025)	Hyperspectral imaging	–
Ground Unmanned Vehicle Robot Arm	(Amertet and Gebresenbet, 2025)	Tracking	Self-trigger control
	(Zhang et al., 2025)	Path planning	HDBD control
	(Xu et al., 2025)	Trajectory	DQN control
	(Guan et al., 2025)	–	Fuzzy PID
	(Liu et al., 2025)	Kinematics	Fuzzy PID
	(Taddei Dalla Torre et al., 2025)	Sensor feedback	Sensor control
Below ground	(Sahu et al., 2025)	–	Microcontroller
	(Holtorf et al., 2023)	Low-power comm.	–
	(Bayrakdar, 2019)	–	Threshold control

### 3.1 Agricultural air-ground cooperative communication and sensing

Reliable cooperative communication is the fundamental question for heterogeneous agricultural robot collaboration. However, agricultural air–ground systems face a communication-constrained cooperative autonomy challenge. In such systems, unreliable wireless links, intermittent connectivity, limited bandwidth, latency constraints, and constrained onboard energy and computation resources jointly limit cooperative perception, localization, path planning, resource allocation, and field operation control. For example, vegetation occlusion, complex terrain and large-scale spatial distribution in agricultural scenarios make this problem more challenging than general industrial scenarios.

**Figure 5:**
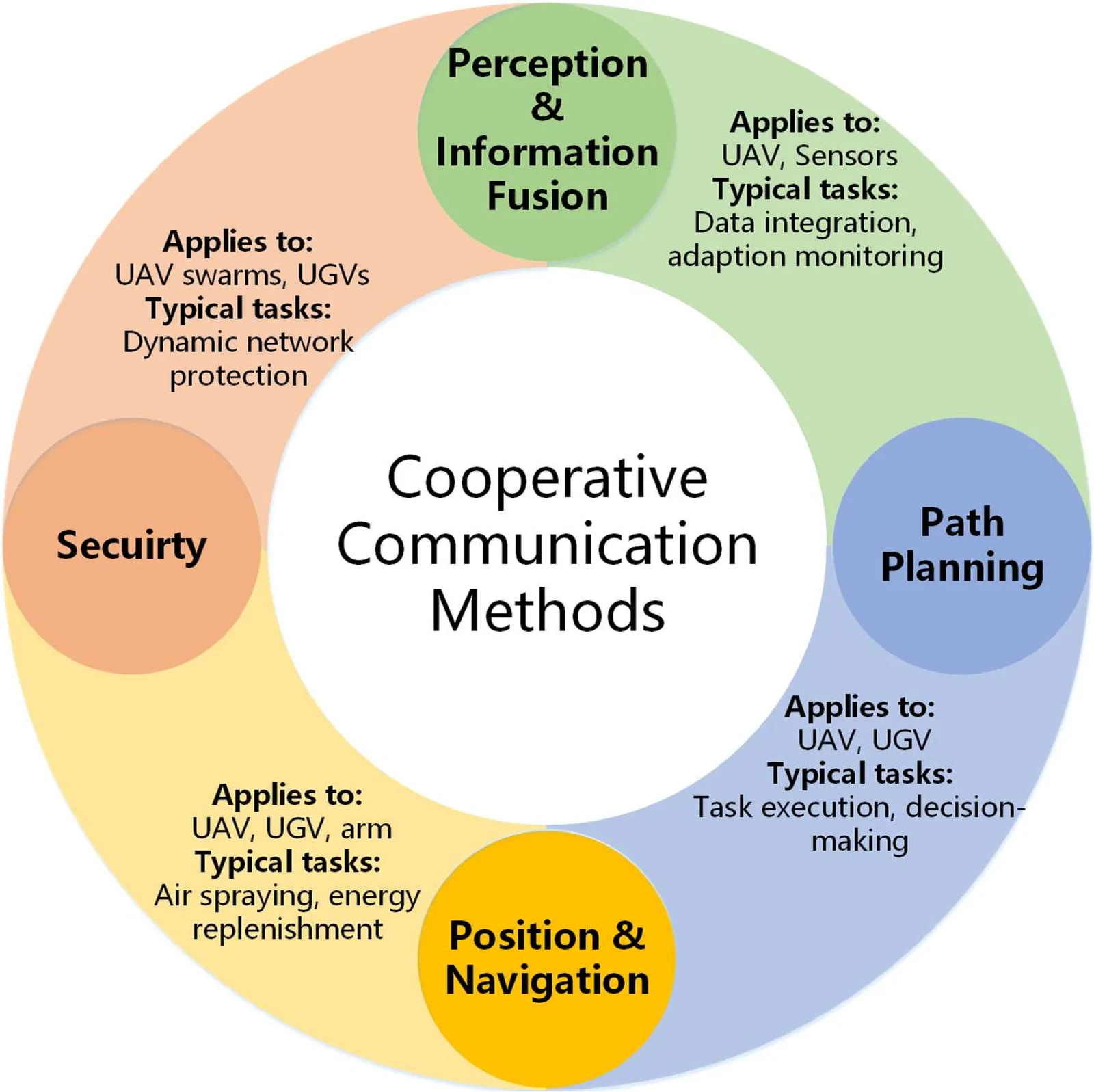
Comparison of cooperative communication methods for heterogeneous agricultural robot agents.

To begin with, a major research direction focuses on using aerial–ground links to support cooperative perception and information fusion. For example, as is shown in **Figure 5**, visual communication interaction allows UAVs to guide UGVs through real-time tracking and motion control (Wang and Cao, 2025). Wi-Fi–based links enable UAVs to collect data from ground nodes, combining aerial remote sensing with soil information to form multi-modal agricultural datasets (Li, 2024). However, dynamic agricultural and outdoor environments may cause link instability, which means that adaptive multi-link communication architectures are required to ensure reliable coordination. Besides, distributed sensor networks integrated with UAV and UGV systems rely heavily on adaptive environment monitoring for adaptive soil analysis, fertilization, and irrigation (Ciacco et al., 2025), which demonstrates that communication is the core enabler of synchronized perception, transforming independent agents into an integrated sensing network, and to improve the adaption into the complex environments and to improve the utility in the agricultural system. Thus, cooperative perception in agricultural environments is highly communication-constrained, which makes it difficult to maintain timely and reliable multi-source information fusion under agricultural environment.

Building on communication-constrained cooperative perception, another related challenge lies in communication-aware path planning, localization because of the constrained resource allocation requirements. Many studies investigate cooperative path planning and localization where the impact of communication transmission quality directly influences system decision-making and resource allocation. On the one hand, such cooperation is fundamentally challenged by limited bandwidth, latency constraints, and the restricted energy and computation resources of UAVs and UGVs. For example, formation tracking strategies depend on distributed communication among heterogeneous robots to maintain time-varying formations and avoid collisions (Golchin et al., 2025). Large-scale coverage frameworks incorporate communication distance and endurance limits into region segmentation and UAV–UGV allocation (Wu et al., 2025; Zhang et al., 2025). In (Sun et al., 2025), a hybrid MADDPG–PSO multi-agent learning and optimization approach was proposed, which explicitly balance coverage maximization with communication stability requirements and overcome the resource allocation problem. Authors in (Sun et al., 2019) investigated an adaptive learning-based task offloading scheme for vehicular edge computing systems, where a multi-armed bandit approach is employed to dynamically select edge servers and minimize task execution latency. These studies indicate that communication cooperation methods play an important role in supporting cooperative planning, decision-making, and control modules, while achieving robust cooperative decision-making under uncertain communication conditions and limited edge resources remains a challenging open problem.

Meanwhile, communication is indispensable for supporting collaborative field operations and localization tasks. Air ground spraying and agricultural manipulation systems rely on stable communication to synchronize UAV navigation, UGV refilling actions, and safety control (Li et al., 2023; Rodrigues Barbosa Júnior et al., 2025). Hybrid exploration and mapping systems require continuous UAV and UGV information exchange to manage energy replenishment, mapping tasks, and navigation accuracy (Bai et al., 2025; Tao et al., 2025). In addition, communication signals themselves can support localization, where UAV-transmitted signals and UGV mounted ULAs enable DOA-based cooperative positioning, and the NODGA-CS model improves accuracy under limited snapshots (Zhang et al., 2025). These studies show that communication-assisted agricultural localization has not yet been fully investigated under different channel uncertainty and mobility-induced environments. In particular, when communication delay or packet loss occurs, localization errors may accumulate and further affect navigation, refilling, manipulation, and safety-control decisions.

Finally, ensuring secure communication remains a fundamental challenge for multi-drone agricultural systems. As highlighted in (Charfi et al., 2025), agricultural multi-UAV networks are exposed to multi-layer security threats, including spanning sensing, hardware, software, and, most critically, wireless communication links. The open and shared nature of wireless channels makes heterogeneous UAV–UGV swarms particularly vulnerable to denial-of-service attacks, jamming, spoofing, and other cyber–physical threats, which can severely disrupt coordination and mission execution. Although cloud–fog cooperative architectures have been explored to enhance network resilience and achieve distributed consensus under adversarial conditions (Li et al., 2024), maintaining secure, low-latency, and resource-efficient communication in highly dynamic air–ground environments remains challenging. These studies collectively reveal that communication security is not merely a technical enhancement but a critical bottleneck that fundamentally limits the reliability, trustworthiness, and scalability of collaborative agricultural robotic systems.

Therefore, in practical agricultural scenarios with vegetation occlusion, uneven terrain, mobile robots, and limited onboard resources, how to maintain reliable communication links, reliable information sharing, reliable cooperative decision-making, and reliable field operation execution remains an open challenge.

### 3.2 Heterogeneous agricultural robots control design

The core difficulty of heterogeneous agricultural robot control lies in the need to simultaneously satisfy accuracy, real-time performance and safety under dynamic, unstructured and uncertain environments, which is a multi-constrained coupled optimization problem.

With the rapid development of smart agriculture, multi-UAV systems are increasingly deployed for large-scale field operations, transportation, data collection, and monitoring. Collaborative control of multi-UAV groups relies heavily on efficient path planning and obstacle-avoidance algorithms. Existing algorithms summarized in (Wang et al., 2024) and (Mustafa and Kakshar, 2025) mainly fall into two categories: centralized and distributed frameworks. Centralized approaches such as Rapidly-exploring Random Tree (RRT) and improved APF provide strong global optimization but lack scalability, while distributed approaches including greedy strategies and dynamic programming enhance robustness through local communication. Future development requires combining AI and multi-sensor fusion to handle complex agricultural tasks. In addition, hybrid approaches continue to emerge. (Ma et al., 2025) proposes a DSA-AAPF framework that improves APF by introducing adaptive gravitational gain and speed tuning, overcoming issues of excessive long-range attraction and insufficient near-target pull, achieving smooth trajectories and fast convergence. (Guo et al., 2025) introduces a two-level hybrid system where global path planning uses improved APF and local optimization employs DQN reinforcement learning, along with distributed coordination for multi-UAV conflict resolution. In orchard scenarios (Tang et al., 2025), presents EN-MASCA, integrating a virtual navigator and deep RL for UAV swarm patrolling, significantly improving path deviation and formation consistency. Decentralized coordination was further studied in (Lai et al., 2025), where UAVs adopt reference-following and autonomous role allocation for scalable formation control. The concept of MARL for cooperative UAV groups was emphasized in (Gowda, 2025), enabling decentralized learning-based coordination in complex agricultural environments. Multi-UAV collaborative transportation was examined in (Rizaldi and Kim, 2025) through heterogeneous UAV grouping and dimensionality reduction for real-time optimization, and (Chen et al., 2024) applies disturbance-observer-based control for stable payload transport.

Real-time safe planning is addressed by (Rubinacci et al., 2025), which uses recursive feasibility, positive-definiteness certificates, and convex–concave optimization to produce collision-free online trajectories. For wireless sensing and charging applications (Xu et al., 2024), uses improved k-means for load-balanced UAV clustering and models charging routes as a weighted TSP, reducing energy loss and node failure. To ensure safe multi-UAV operations (Alqudsi and Makaraci, 2025), proposes SARG, a dual-avoidance framework integrating D* Lite for synchronized path planning under dynamic constraints. Mapless navigation using deep adaptive learning is introduced in (Mo et al., 2024), where monocular vision replaces SLAM in multi-UAV cooperative indoor tasks. Communication optimization for UAV groups is addressed in (Ahmad et al., 2025), which adopts NOMA-based distributed resource allocation to enhance spectral efficiency with low computational complexity.

To provide a structured overview of the above developments, the representative cooperative control strategies discussed in this section are further synthesized and categorized according to their typical agricultural tasks, system-level advantages, limitations, and applicable robotic platforms. In particular, beyond multi-UAV coordination, heterogeneous agricultural operations increasingly involve the integration of UAVs, ground vehicles, manipulators, and subsurface agents, which requires control architectures that can simultaneously address scalability, robustness, and cross-platform interoperability. As summarized in **Table 3**, representative cooperative control strategies are categorized according to their control architecture and application characteristics. Therefore, this section summarizes recent real agricultural studies from the perspective of cooperative control design, highlighting how different planning, learning-based, perception-driven, and task-allocation approaches support heterogeneous agricultural robot systems under practical field constraints.

**Table 3:** Representative agricultural robot control and coordination studies by source type, system class, and validation conditions.

Ref	Study type	Method or system	Application	Platform and system class	Validation type	Enironment	Main contribution	Main limitation
(Ju et al., 2022)	Review	Multi-robot architectures and coordination	Farm-scale autonomous operations	UAV/UGV systems; homogeneous and heterogeneous	Literature review	Various agricultural settings	Reviews architectures, coordination, and key challenges	No quantitative control benchmark
(Wang et al., 2023)	Algorithm study	CO-MAMSY path planning and task allocation	Multi-machine field harvesting	Machine fleet; homogeneous multi-robot	Simulation and map case	Mapped open field	Jointly optimizes routes and task allocation	Centralized and not physically validated
(Huang et al., 2023)	Algorithm study	Task assignment and coverage planning	Cooperative pesticide spraying	UAV swarm; homogeneous multi-robot	Simulation and flight tests	Simulated area and outdoor site	Integrates task allocation and collision-free coverage	Dynamic environments remain simplified
(Yu et al., 2023)	Learning control	Double-DQN obstacle avoidance	Dynamic obstacle avoidance	Single UGV	Training, simulation, and field tests	Farmland	Improves adaptability in unknown environments	No formal safety guarantee
(He et al., 2022)	Control study	Pose-corrected MPC tracking	Machinery navigation and tracking	Single agricultural UGV	Field tests	Paddy field	Improves tracking after abrupt pose errors	Model-dependent and computationally demanding
(Jing et al., 2023)	System prototype	GNSS/INS land-levelling control	Precision land levelling	Tractor–scraper; integrated platform	Prototype and field tests	Open farmland	Improves positioning, tracking, and levelling	Limited by GNSS availability
(Zhang et al., 2022)	Control algorithm	NFTSM with disturbance observer	Tractor tracking under disturbances	Single agricultural tractor	Simulink/CarSim co-simulation	Simulated trajectories	Provides finite-time convergence and disturbance rejection	No physical field validation; possible chattering
(Chen et al., 2024)	Field prototype	Dynamic visual-servo harvesting	Continuous fruit harvesting	UGV + manipulator; mobile manipulator	Prototype and real-world tests	Orchard	Coordinates localization, motion, and picking	Sensitive to lighting and occlusion
(Jin and Han, 2024)	Review	Precision-agriculture robotic arms	Harvesting, pruning, and pollination	Robotic arms/mobile bases; mainly single-platform	Literature review	Greenhouses, fields, and orchards	Reviews hardware, perception, planning, and applications	No unified control or validation benchmark
(Klopfenstein and Lussier Desbiens, 2024)	Prototype study	Terra-22 aerial soil sampler	Autonomous soil sampling	UAV + sampling tool; single platform	Prototype and field tests	Compacted field	Enables rapid aerial soil sampling	Limited sampling depth and payload
(Ren et al., 2024)	Review	UAV–UGV collaboration review	Industrialized agriculture	UAV/UGV systems; single and heterogeneous	Literature review	Various agricultural settings	Compares aerial, ground, and cooperative modes	No common quantitative benchmark
(Chen et al., 2025)	Field prototype	UAVs–UGV boom sprayer	Cooperative boom spraying	UAV swarm + UGV; heterogeneous	Prototype and field tests	Field spraying	Extends spray width through coordinated operation	Requires accurate synchronization and communication
(Mammarella et al., 2022a)	Theoretical framework	Air–ground guidance and mission planning	Agriculture 4.0 missions	UAV + UGV; heterogeneous	Analytical and model-based	Generic agricultural missions	Provides a unified cooperation framework	No direct field validation
(Mammarella et al., 2022b)	Case study	UAV mapping and UGV navigation	Cooperative vineyard operation	UAV + UGV; heterogeneous	Simulation and field tests	Sloped vineyard	Demonstrates mapping-to-navigation operation	Scenario-specific and small-scale
(Chen et al., 2024)	Field prototype	LiDAR-based targeted spraying	Orchard spraying	UAV + UGV; heterogeneous	Prototype and orchard trials	Honey pomelo orchard	Links aerial profiling with ground spraying	Communication-dependent; limited multi-site validation

Multi-UAV control in agricultural scenarios faces several core challenges:

1. formation stability constraints: maintaining tight formations under time-varying topologies, packet loss, and communication delays; centralized controllers suffer scalability limits while purely local schemes risk inconsistency. Robust consensus and decentralized reference-following frameworks are required to tolerate switching graphs and delays.
2. real-time safe navigation and obstacle avoidance: agricultural environments are cluttered and dynamic (trees, terrain, workers), requiring fast replanning with collision guarantees; incremental planners (e.g., D* Lite) and convex/convex-concave optimization with recursive feasibility are effective for on-line safe re-planning.
3. energy and endurance constraints: limited battery life forces joint planning of tasks, trajectories and recharging/rotation strategies to maximize mission utility; multi-UAV charging/clustered routing and rolling-horizon energy-aware allocation have been proposed.
4. Current algorithms are mostly validated in simulation or simple environments, and their robustness and real-time performance in real complex agricultural environments with dynamic obstacles still need to be further verified.

## 4 Future trends and developments

Smart agriculture is increasingly shaped by heterogeneous collaboration across communication, sensing, computation, and control. Integrated communication–sensing architectures, together with edge/fog computing and UAV-assisted networks, provide scalable and low-latency support for large-scale agricultural IoT systems. At the robotic level, dual-arm and multi-arm platforms demonstrate the benefits of tightly coupled perception, control, and task allocation in improving efficiency and safety. At the system level, heterogeneous multi-robot collaboration relies on hierarchical control, unified task representations, and robust fault-tolerance mechanisms to enable flexible and resilient full-cycle agricultural operations. Collectively, these developments outline a unified technical framework for next-generation intelligent agricultural systems.

### 4.1 Integrated communication and sensing for heterogeneous agriculture

Given the communication reliability challenges identified in Section 3.1, integrated communication and sensing is regarded as a key technical direction to solve the bottleneck of cooperative perception 560 for heterogeneous agricultural systems. The main investigation of this paper is focus on the agricultural 561 robot-oriented scenarios, such as greenhouse monitoring, orchard inspection, UAV–UGV cooperative 562 spraying, crop phenotyping, and field-scale plant health assessment, rather than general-purpose IoT 563 networking.

From the perspective of communication–sensing cooperation, the future heterogeneous agriculture will involve tightly coupled sensing, communication, and control loops among UAVs, UGVs, in-field sensors, irrigation/fertilization actuators, and farm management platforms. Strict latency requirements, and reliable interoperability are required not only for data exchange, but also for time-sensitive agricultural operations, including crop growth monitoring, disease and pest detection, precision spraying, autonomous harvesting, and cooperative localization in orchards or greenhouses.For example, to enhance crop yields and mitigate potential natural disasters, comprehensive sensing capabilities are indispensable. These include satellite imagery, weather monitoring, and in-field sensor data collection, which together support agricultural monitoring and planting task design. Furthermore, the communication capabilities of agricultural devices play a vital role in improving link capacity and interoperability among heterogeneous Internet-of-Things (IoT) devices. In (Morchid et al., 2024), the application of sensors in agricultural communication systems is investigated, including data exchange among weather monitoring, irrigation, and fertilizer sensors. Additionally, a novel IoT cloud-based communication framework is proposed in (Sarpal et al., 2022), where an Android application is developed to assist farmers with weather prediction and crop recommendations. Besides, the authors in (Avşar and Mowla, 2022) explore five different wireless communication protocols for IoT-based agricultural applications. Agricultural IoT systems often consist of sensors and actuators from multiple vendors, each employing proprietary data formats and protocol stacks, which leads to fragmented communication and difficulty in achieving seamless coordination among devices. This heterogeneity not only increases integration complexity but also reduces data consistency, inhibits scalable deployment that prevent efficient cross-platform information exchange and collaboration. As illustrated in (Garlando et al., 2020), deploying sensor technologies along with appropriate communication protocols constitutes a direct and effective approach to optimizing agricultural processes. Herein, communication and sensing protocol interoperability is identified as a critical requirement for enabling seamless coordination across heterogeneous agricultural platforms.

For agricultural robots, such interoperability directly affects whether perception results from plant sensors and UAV imagery can be reliably transformed into executable field operations, such as path planning, variable-rate spraying, irrigation control, and targeted crop monitoring. This architecture can effectively reduce the deployment cost of agricultural IoT nodes and improve the coverage of sensing data in large-scale orchards, greenhouses, and farmlands.

Besides, the paradigms of edge and fog computing are expected to be instrumental in facilitating UAV-centric heterogeneous systems to satisfy the rigorous demands of trajectory planning, localization, and real-time decision-making. On the one hand, agricultural environments are often characterized by limited terrestrial infrastructure, vast geographic coverage, and intermittent connectivity, which make purely ground-based computing and communication solutions insufficient. As a result, hybrid architectures that integrate low-power wide-area networks (LPWANs), cellular networks (e.g., 5G), satellites, and UAV-mounted communication nodes are expected to become a dominant trend. By deploying AI-enabled edge or fog computing capabilities on UAVs or UAV-associated gateways, sensing data can be pre-processed, filtered, and fused in real time before being forwarded to higher layers or cloud platforms.

In such architectures, UAVs not only act as mobile communication relays but also serve as airborne computing platforms that collect, aggregate, and process sensing data from distributed ground devices. On the other hand, driven by labor shortages in rural regions and the harsh operating conditions associated with specific crops, UAV-enabled communication and sensing are further required to support adaptive trajectory planning and cooperative localization for ground robots and sensors. Offloading sensing data and intermediate localization information to nearby UAVs allows computationally demanding tasks like trajectory optimization, map updating, and position estimation to be conducted in proximity to the data sources. This effectively decreases end-to-end latency and strengthens system responsiveness. This highlights the growing importance of UAVs as both communication and computation hubs in heterogeneous agricultural systems.

In such architectures, UAVs not only act as mobile communication relays but also serve as airborne computing platforms that collect, aggregate, and process sensing data from distributed ground devices. In areas without stable ground communication infrastructure, UAV can also provide real-time decision support for ground agricultural robots, such as UGVs performing spraying, inspection, refilling, and harvesting operations.

Finally, security mechanisms in heterogeneous smart agricultural systems are expected to shift toward physical-layer security (PLS), especially in UAV-centric air–ground networks. Traditional cryptographic approaches mainly rely on computational complexity and key management, whose long-term robustness is increasingly challenged by the rapid advancement of computing capabilities and the emergence of quantum-enabled attacks. Moreover, the dynamic topology and resource-constrained nature of UAV-assisted agricultural networks make frequent key distribution and management costly and inefficient. By contrast, physical-layer security exploits the inherent randomness of wireless channels, such as channel fading, spatial diversity, and noise, to provide information-theoretic security guarantees without relying on computational hardness. In UAV-assisted agricultural systems, mobility-induced channel variations, three-dimensional spatial diversity, and controllable UAV trajectories can be deliberately leveraged to enhance secrecy performance.

Therefore, physical-layer security can serve as a complementary low-cost security mechanism for resource-constrained agricultural sensor nodes, underground devices, and UAV-UGV cooperative links.

### 4.2 Collaboration between isomorphic dual-arm and multi-arm agricultural robots

In view of the low efficiency and high collision risk of single-arm operation summarized in Section 2, dual-arm and multi-arm collaborative robots have broad application prospects in structured agricultural environments such as greenhouses. Compared to single-arm systems, dual-arm collaboration can simulate the coordinated operation of human hands, enhancing task adaptability and safety while improving operational efficiency.

The core capabilities of dual-arm collaboration are manifested at three levels: perception, control, and task allocation. In terms of perception, the DigiHortiRobot dual-arm system proposed by Fernandez et al. In (Fernández et al., 2025), crop-structure perception and virtual modeling are used to support operation strategy adjustment, thereby improving the adaptability of dual-arm agricultural robots to different plant growth states and reducing repetitive manual programming. Similarly, Ishibashi et al. (2025) combined Mask R-CNN visual recognition with a hydraulic soft-bodied robotic arm to achieve non-destructive handling of crops under high loads, highlighting the importance of perception-execution integration.

In terms of motion control and safe collaboration, Liu et al. (2025) proposed a collision avoidance strategy based on relative velocity, embedding obstacle avoidance constraints into the null space of the task space, thus achieving dynamic collision avoidance for dual-arm systems while ensuring the task accuracy of the end-effector. This method provides theoretical support for safe collaboration in dense work environments.

Task allocation and scheduling are crucial for efficient dual-arm collaboration. Park and Son (2024) constructed a visual understanding framework based on scene graphs, incorporating an attribute-weighted priority model (considering fruit size, maturity, and accessibility) to dynamically allocate targets to the left and right arms. Yan and Li (2025) divided the working area with a boundary of x=0 and adopted the “short-distance first” rule to optimize the apple picking sequence, effectively reducing interference between the arms. He et al. (2022) further introduced Brain Storm Optimization (BSO) and K-means clustering to spatially partition kiwifruits, and generated the optimal picking path based on the Multiple Traveling Salesperson Problem (MTSP), demonstrating the value of combinatorial optimization in task planning. As system complexity increases, multi-arm collaboration (or extended dual-arm systems) poses higher demands on intelligent scheduling. Guo et al. (2025) modeled the harvesting process as a Markov decision process and utilized an LSTM-PPO deep reinforcement learning architecture to capture the temporal dependencies between fruit distribution, robot state, and task switching, enabling dynamic task replanning. Zhu et al. (2025) employed mixed integer linear programming (MILP) to optimize the “stop-harvest” strategy for strawberry robots, minimizing the total harvesting time while ensuring pose coverage and structural collision avoidance, demonstrating the applicability of operations research methods in precision agriculture.

To enhance task continuity and system throughput, Zhang et al. (2025) proposed a parallel dual-arm control paradigm. This paradigm employs an asynchronous collaborative mechanism of “move-observe-harvest” to first divide independent task areas to reduce coupling interference, and then implements load balancing scheduling based on the Shortest Time First First-Seen Harvest (ST-FSH) principle. This method achieves spatiotemporal synchronization of visual detection, motion control, and harvesting actions, significantly reducing task interruptions and task misalignments.

In summary, the current dual-arm agricultural robots have progressed from basic collaboration to a new stage characterized by the deep integration of intelligent perception, safety control, and optimized scheduling. When expanding to multi-arm systems in the future, challenges such as communication synchronization, global resource allocation, heterogeneous arm coordination, and large-scale real-time decision-making need to be further addressed. Combining learning-driven and model-driven methods to construct a scalable, robust, and efficient multi-arm collaboration framework will become a key direction for promoting the large-scale application of agricultural robots.

### 4.3 Heterogeneous agricultural robot collaborative control

To address the scalability and fault tolerance challenges of heterogeneous multi-robot systems, future cooperative control frameworks need to develop towards a hierarchical and distributed architecture.

Modern agricultural production operates as a complex, large-scale cyber–physical system characterized by high task density, dynamic spatiotemporal variability, and strict requirements on real-time responsiveness, operational precision, and system continuity. Although single-function robots have demonstrated effectiveness in isolated tasks such as targeted pesticide application or crop maturity recognition, their overall utility remains inherently constrained by limited mobility speed, narrow task scope, finite energy endurance, and insufficient adaptability to heterogeneous and time-varying field conditions. These limitations make such platforms poorly suited for the holistic, full-cycle coordination demanded by agricultural workflows, encompassing seeding, environmental monitoring, irrigation, harvesting, disease forecasting, and post-harvest logistics.

Multi-robot swarm collaboration has emerged as a foundational paradigm in intelligent agriculture, specifically designed to address these pressing challenges. This shift reflects not merely an increase in hardware scale, but a strategic transition toward resilient, adaptive, and intelligent system-level management.

The effectiveness of this paradigm relies on the coordinated advancement of three tightly coupled technical pillars: intelligent task scheduling, efficient and reliable communication architectures, and seamless integration of heterogeneous robotic platforms.

From a system architecture perspective, agricultural control strategies are evolving from rigid centralized schemes toward hybrid intelligence frameworks. Early implementations typically employed a monolithic central controller responsible for global path planning and task allocation, a design prone to computational bottlenecks and single points of failure. Contemporary approaches instead favor hierarchical coordination architectures, in which a supervisory node maintains global objectives and a shared task pool, while individual robots exploit local sensing and peer-to-peer communication to make context-aware decisions. Within this framework, lightweight local auction mechanisms enable robots to dynamically compete for subtasks based on spatial proximity, functional capability, and current workload. A minimal global coordinator intervenes only when necessary, such as in cases of workspace overlap or resource contention, thereby preserving system efficiency without undermining agent autonomy.

Achieving true interoperability within such systems requires more than efficient scheduling mechanisms; it necessitates the elimination of information silos across functionally diverse robotic agents, including aerial platforms for large-scale sensing, ground vehicles for field operations, and manipulators for harvesting and handling tasks. To this end, a universal task description language, typically implemented using extensible formats such as XML or JSON, is increasingly adopted as a common interface for swarm coordination. This unified representation standardizes task semantics, including task type, urgency level, spatial constraints, and resource requirements, while also harmonizing the exchange of sensor data, control commands, and status information. Such standardization facilitates plug-and-play integration of new agents and significantly reduces system deployment and maintenance complexity.

Robustness under uncertainty is further enhanced through fault-tolerant mechanisms based on the concept of a virtual surrogate. Each physical robot maintains a lightweight digital counterpart, hosted on edge or cloud infrastructure, which continuously broadcasts key operational states such as remaining energy, localization confidence, task queue status, and functional health indicators. When a robot experiences partial or complete failure, neighboring agents detect anomalies through heartbeat loss or state inconsistency and, via local consensus protocols, autonomously redistribute tasks, renegotiate operational boundaries, and reassign pending objectives. This decentralized recovery process ensures sustained system performance with minimal human intervention.

Collectively, these advances elevate multi-robot swarms from isolated automation tools to adaptive, self-organizing control substrates for next-generation agricultural systems, enabling high-throughput, low-risk, and resource-efficient production across diverse and dynamically changing farming environments.

## 5 Conclusion

**Figure 6:**
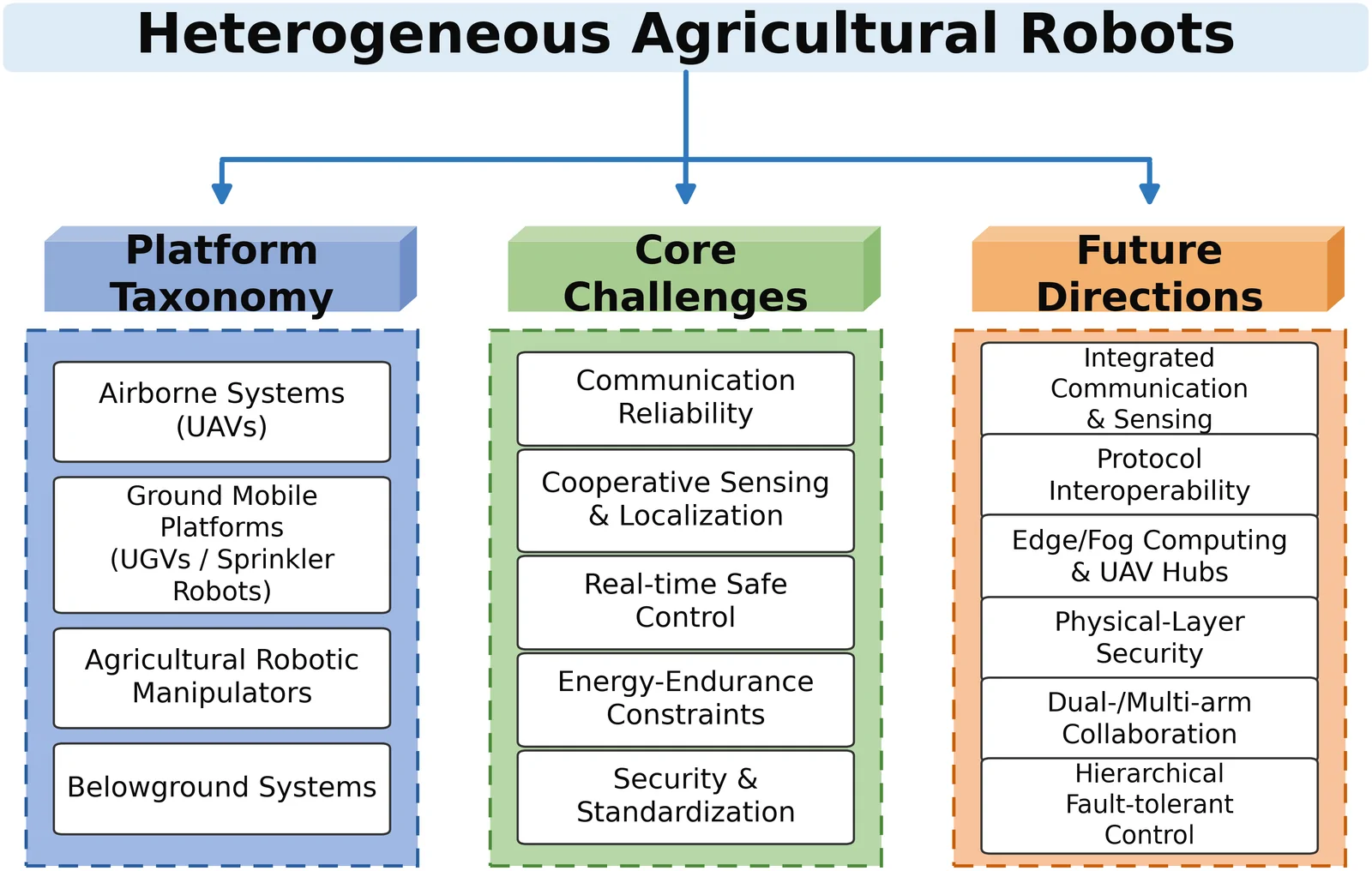
Type, challenges and future trends for heterogeneous agricultural robots.

As is shown in **Figure 6**, this paper has presented a systematic review of collaborative sensing and control technologies for heterogeneous agricultural robots, with a particular focus on recent advances in intelligent control, multi-robot cooperation, and heterogeneous system integration. By analyzing representative platforms, including sprinkler robots, unmanned ground vehicles, unmanned aerial vehicles, robotic arms, and underground robots, we have highlighted how diverse robotic agents contribute complementary capabilities to modern precision agriculture.

The review demonstrates that the evolution of agricultural robotics is shifting from single-function automation toward coordinated multi-agent systems capable of operating across air, ground, and underground domains. Advances in adaptive control, artificial intelligence, visual perception, and multi-sensor fusion have significantly improved autonomy and task performance. At the same time, emerging air–ground–underground integrated architectures offer new opportunities for full-space perception, efficient resource utilization, and resilient operation in complex agricultural environments.

However, several challenges remain unresolved. Reliable cooperative communication, real-time safe control under dynamic and uncertain conditions, energy-aware task scheduling, and system security continue to limit large-scale deployment. Moreover, the increasing heterogeneity of robotic platforms introduces new demands for standardized task descriptions, robust coordination mechanisms, and fault-tolerant system designs.

Looking forward, future agricultural robot systems are expected to evolve toward deeper integration of communication and sensing, intelligent dual-arm and multi-arm collaboration, and scalable heterogeneous multi-robot cooperative control frameworks. By combining learning-driven and model-driven approaches, and by leveraging edge computing and secure communication technologies, Heterogeneous agricultural robot swarms have the potential to become a foundational infrastructure for next-generation smart agriculture. This review provides a consolidated technical perspective and serves as a reference to guide future research and practical applications in this rapidly developing field.

## References

[B47] Abdelhamid M. A. Abdelkader T. K. Sayed H. A. A. Zhang Z. Zhao X. Atia M. F. (2025). Design and evaluation of a solar powered smart irrigation system for sustainable urban agriculture. Sci. Rep. 15, 11761. doi: 10.1038/s41598-025-94251-3 40189625 PMC11973206

[B103] Ahmad S. Ul Abideen S. Z. Kamal M. M. Al-Khasawneh M. A. Issa G. F. Ullah N. . (2025). Resource management for multi-drone communications in next-generation noma-enabled wireless networks. Sci. Rep. 15, 23585. doi: 10.1038/s41598-025-09459-0 40603528 PMC12222829

[B26] Akhil P. Dinesh Kumar P. Lakshmi Priya S . (2025). “ Automated pest classification of paddy field using drone imagery”, in: 2025 8th International Conference on Trends in Electronics and Informatics (ICOEI) ( IEEE), 756–761.

[B101] Alqudsi Y. Makaraci M. (2025). Towards optimal guidance of autonomous swarm drones in dynamic constrained environments. Expert Syst. 42, e70067. doi: 10.1111/exsy.70067 40046247

[B33] Amertet S. Gebresenbet G. (2025). Collision avoidance for wheeled mobile robots in smart agricultural systems using control barrier function quadratic programming. Appl. Sci. 15, 2450. doi: 10.3390/app15052450 30654563

[B19] Amertet S. Gebresenbet G. Alwan H. M. (2024). Modeling of unmanned aerial vehicles for smart agriculture systems using hybrid fuzzy pid controllers. Appl. Sci. 14, 3458. doi: 10.3390/app14083458 30654563

[B62] Amisha, Kumari B. Priya, Kumari S. Srividya B. V. (2023). “ Robotic orchard: A smart and efficient solution for fruit harvesting”, in: 2023 International Conference on Smart Systems for applications in Electrical Sciences (ICSSES) (Piscataway, NJ, USA: IEEE), 1–7.

[B121] Avşar E. Mowla M. N. (2022). Wireless communication protocols in smart agriculture: A review on applications, challenges and future trends. Ad Hoc Networks 136, 102982. doi: 10.1016/j.adhoc.2022.102982

[B85] Bai D. Zhang Z. Li Z. Tang H. Tang C. (2025). “ Bs-hop: A collaborative exploration approach for power-switchable air-ground robots under multiple map structures”, in: 2025 IEEE International Conference on Real-time Computing and Robotics (RCAR) (Piscataway, NJ, USA: IEEE), 576–581.

[B5] Barbosa Junior M. R. Garcia dos Santos R. de Azevedo Sales L. de Oliveira L. P. (2024). Advancements in agricultural ground robots for specialty crops: an overview of innovations, challenges, and prospects. Plants 13, 3372. doi: 10.3390/plants13233372 39683165 PMC11644308

[B58] Barcelos de Assis J. P. Günther G. A. Pellenz M. E. Teixeira M. S. (2025). “ Computer vision and machine learning-based control for a 6-degree-of-freedom robotic arm”, in: 2025 IEEE International Conference on Industrial Technology (ICIT) (Piscataway, NJ, USA: IEEE), 1–6.

[B69] Bayrakdar M. E. (2019). A smart insect pest detection technique with qualified underground wireless sensor nodes for precision agriculture. IEEE Sens. J. 19, 10892–10897. doi: 10.1109/jsen.2019.2931816 25079929

[B30] Ben Miled M. Grande D. Li X. Liu Y. (2023). “ Exploring the technical advances and limits of autonomous uavs for precise agriculture in constrained environments”, in: 2023 IEEE 19th International Conference on Automation Science and Engineering (CASE) (Piscataway, NJ, USA: IEEE), 1–8.

[B21] Borah S. K. Padhiary M. Sethi L. N. Kumar A. Saikia P. (2025). Precision farming with drone sprayers: A review of auto navigation and vision-basedoptimization. J. Biosyst. Eng. 50, 1–19. doi: 10.1007/s42853-025-00263-2

[B41] Cai Y. Cao Y. (2025). Positional tracking study of greenhouse mobile robot based on improved monodepth2. IEEE Access. 13, 106690–106702. doi: 10.1109/access.2025.3578135 25079929

[B67] Chang C.-L. Huang C.-C. (2024). Design and implementation of an ai-based robotic arm for strawberry harvesting. Agriculture 14, 2057. doi: 10.3390/agriculture14112057 30654563

[B88] Charfi A. Ayed S. Chaari L. (2025). “ Drone security in connected agriculture: Threats, datasets, and ai-driven solutions”, in: 2025 12th IFIP International Conference on New Technologies, Mobility and Security (NTMS) (Piscataway, NJ, USA: IEEE), 26–33.

[B111] Chen M. Chen Z. Luo L. Tang Y. Cheng J. Wei H. . (2024). Dynamic visual servo control methods for continuous operation of a fruit harvesting robot working throughout an orchard. Comput. Electron. Agric. 219, 108774. doi: 10.1016/j.compag.2024.108774 38826717

[B98] Chen W.-C. Kawanishi M. Lu C.-Y. Ciou S.-Y. Lin C.-L. Hsu C. H. (2024). Cooperative load transportation of multi-drones based on disturbance observer and formation control. IEEE Access 12, 77393–77405. doi: 10.1109/access.2024.3406677 25079929

[B118] Chen Y. L. Liu Z. B. Lin Z. Xu Z. F. Guan X. L. Zhou Z. Y. . (2024). Uav-ugv cooperative targeted spraying system for honey pomelo orchard. Int. J. Agric. Biol. Eng. 17, 22–31. doi: 10.2139/ssrn.4725158

[B115] Chen Y. Liu Z. Xu Z. Lin J. Guan X. Zhou Z. . (2025). Uavs-ugv cooperative boom sprayer system based on swarm control. Comput. Electron. Agric. 235, 110339. doi: 10.1016/j.compag.2025.110339 38826717

[B66] Cheng S. Yu Z. Li Z. Fan Q. Lyu S. Wen W. . (2025). Design and analysis of a lightweight redundant-degree-of-freedom fruit-picking robot arm. J. Field Rob. 42, 2815–2825. doi: 10.1002/rob.22545 41531421

[B77] Ciacco A. Giallombardo G. Guerriero F. Saccomanno F. P. (2025). “ Monitoring agricultural fields through collaboration between autonomous robots and drones”, in: 2025 IEEE Conference on Technologies for Sustainability (SusTech) (Piscataway, NJ, USA: IEEE), 1–8.

[B32] Comparetti A. Fagiolini A. Fountas S. Cascio V. (2025). Field robots for precision agriculture. AIMS Agric. Food 10, 885–916. doi: 10.3934/agrfood.2025046

[B59] Dong J. Deng L. Wan D. Liu H. (2024). “ Research and simulation of intelligent grasping of robotic arm based on machine vision recognition”, in: 2024 IEEE 4th International Conference on Power, Electronics and Computer Applications (ICPECA) (Piscataway, NJ, USA: IEEE), 1085–1089.

[B15] Dong H. Ma X. Zhang S. (2025). Multi-flight path planning for a single agricultural drone in a regular farmland area. Sustainability 17, 2433. doi: 10.3390/su17062433 30654563

[B6] Droukas L. Doulgeri Z. Tsakiridis N. L. Triantafyllou D. Kleitsiotis I. Mariolis I. . (2023). A survey of robotic harvesting systems and enabling technologies. J. Intell. Rob. Syst. 107, 21. doi: 10.1007/s10846-022-01793-z 36721646 PMC9881528

[B123] Fernández R. Navas E. Rodríguez-Nieto D. Rodríguez-González A. A. Emmi L. (2025). Digihortirobot: An ai-driven digital twin architecture for hydroponic greenhouse horticulture with dual-arm robotic automation. Future Internet 17, 347. doi: 10.3390/fi17080347

[B46] Furqan M. Lubis S. A. Harahap R. L. (2022). Automatic plant watering system based on air temperature and soil humidity using the fuzzy sugeno method. Sinkron: jurnal Dan. penelitian teknik informatika 6, 2576–2583. doi: 10.33395/sinkron.v7i4.11893

[B122] Garlando U. Bar-On L. Avni A. Shacham-Diamand Y. Demarchi D. (2020). “ Plants and environmental sensors for smart agriculture, an overview”, in: 2020 IEEE SENSORS, Piscataway, NJ, USA: IEEE. 1–4.

[B34] Ghorab M. Lorenzen M. (2025). Multi-waypoint path planning and motion control for non-holonomic mobile robots in agricultural applications. Arxiv Preprint Arxiv:2507.23350. 103–108. doi: 10.34749/3061-0710.2025.17

[B78] Golchin M. Bayat F. Mobayen S. Fekih A. (2025). Adaptive formation tracking control for heterogeneous unmanned air/ground vehicles: A barrier function approach for minimizing energy consumption. Iranian J. Sci. Technology Trans. Electrical Eng. 49, 1–23. doi: 10.1007/s40998-025-00865-8 30311153

[B96] Gowda D. (2025). Multi-agent reinforcement learning for coordinated drone swarms. 11 (2), 3218–3223.

[B28] Guan S. Shimazaki Y. Takahashi K. Kato H. Fukami K. Watanabe S. (2025). Agricultural drone-based variable-rate n application for regulating wheat protein content. Drones 9, 310. doi: 10.3390/drones9040310 30654563

[B42] Guan C. Zhao W. Xu B. Cui Z. Yang Y. Gong Y. (2025). Design and experiment of electric uncrewed transport vehicle for solanaceous vegetables in greenhouse. Agriculture; Basel 15, 118. doi: 10.3390/agriculture15020118 30654563

[B129] Guo Z. Fu H. Wu J. Han W. Huang W. Zheng W. . (2025). Dynamic task planning for multi-arm apple-harvesting robots using lstm-ppo reinforcement learning algorithm. Agriculture 15, 588. doi: 10.3390/agriculture15060588 30654563

[B93] Guo L. Shu X. Zhu Y. (2025). Smart farming with swarm intelligence: Hybrid algorithm for optimal multi drone plant protection paths. Pakistan J. Agric. Sci. 62, 215–225. doi: 10.21162/PAKJAS/25.9225

[B108] He J. Hu L. Wang P. Liu Y. Man Z. Tu T. . (2022). Path tracking control method and performance test based on agricultural machinery pose correction. Comput. Electron. Agric. 200, 107185. doi: 10.1016/j.compag.2022.107185 38826717

[B128] He Z. Ma L. Wang Y. Wei Y. Ding X. Li K. . (2022). Double-arm cooperation and implementing for harvesting kiwifruit. Agriculture 12, 1763. doi: 10.3390/agriculture12111763 30654563

[B68] Holtorf L. Titov I. Daschner F. Gerken M . (2023). UAV-based wireless data collection from underground sensor nodes for precision agriculture. AgriEngineering 5, 338–354. doi: 10.3390/agriengineering5010022 30654563

[B106] Huang J. Luo Y. Quan Q. Wang B. Xue X. Zhang Y. (2023). An autonomous task assignment and decision-making method for coverage path planning of multiple pesticide spraying uavs. Comput. Electron. Agric. 212, 108128. doi: 10.1016/j.compag.2023.108128 38826717

[B124] Ishibashi K. Yamada H. Ishikawa H. Toi H. Azami O. Yamamoto K. . (2025). Dual-arm robotic system with hydraulically-driven soft hand for transportation of large cabbages. Adv. Rob. 39, 1–14. doi: 10.1080/01691864.2025.2512389 37339054

[B22] Jeba Kumar J. S. Yuvaraj S. Gothavari V. Anantharaj R. (2025). “ Automatic pesticide spraying drone”, in: 2025 IEEE International Students’ Conference on Electrical, Electronics and Computer Science (SCEECS) (Piscataway, NJ, USA: IEEE), 1–5.

[B4] Jin T. Han X. (2024). Robotic arms in precision agriculture: A comprehensive review of the technologies, applications, challenges, and future prospects. Comput. Electron. Agric. 221, 108938. doi: 10.1016/j.compag.2024.108938 38826717

[B109] Jing Y. Li Q. Ye W. Liu G. (2023). Development of a GNSS/INS-based automatic navigation land levelling system. Comput. Electron. Agric. 213, 108187. doi: 10.1016/j.compag.2023.108187 38826717

[B104] Ju C. Kim J. Seol J. Son H. I. (2022). A review on multirobot systems in agriculture. Comput. Electron. Agric. 202, 107336. doi: 10.1016/j.compag.2022.107336 38826717

[B64] Kamei T. Wang Z. (2025). “ A non-energized robotic hand using locking mechanism and silicone belts for grasping heavy agricultural products”, in: 2025 IEEE/SICE International Symposium on System Integration (SII) (Piscataway, NJ, USA: IEEE), 429–433.

[B29] Kar D. Dhal S. B. (2025). Advancing food security through drone-based hyperspectral imaging: applications in precision agriculture and post-harvest management. Environ. Monit. Assess. 197, 283. doi: 10.1007/s10661-025-13650-1 39939472

[B53] Khan H. A. Farooq U. Saleem S. R. Rehman U. Tahir M. N. Iqbal T. . (2024). Design and development of machine vision robotic arm for vegetable crops in hydroponics. Smart Agric. Technol. 9, 100628. doi: 10.1016/j.atech.2024.100628 38826717

[B113] Klopfenstein A. Lussier Desbiens A. (2024). Terra-22: An aerial system for soil sampling and analysis. Can. J. Soil Sci. 12, 1–14. doi: 10.1139/dsa-2023-0010

[B44] Krishna A. V. Raj B. S. Praneeth P. Sunil M. L. (2025). Agriculture automatic water sprinkler based on moisture sensor. Indo-American J. Mechanical Eng. 14, 24–29.

[B63] Kulhandjian H. Amely N. Kulhandjian M. (2025). “ Ai-powered fruit harvesting system using a robotic arm for precision agriculture”, in: 2025 International Conference on Computing, Networking and Communications (ICNC) (Piscataway, NJ, USA: IEEE), 505–509.

[B95] Lai C.-H. Lu C.-Y. Lin C.-L. Chen Y.-Y. (2025). Enhancing drone swarm efficiency through a high-flexibility biomimetic formation algorithm. Drone Syst. Appl. 13, 1–26. doi: 10.1139/dsa-2024-0066 34819996

[B24] Laura D. Urrutia E. P. Salazar F. Ureña J. Moreno R. MaChado G. . (2025). Aerial remote sensing system to control pathogens and diseases in broccoli crops with the use of artificial vision. Smart Agric. Technol. 10, 100739. doi: 10.1016/j.atech.2024.100739 38826717

[B76] Li X. (2024). “ Research on agricultural monitoring and progressive map creation in the context of ground-air coordination”, in: Journal of Physics: Conference Series (Bristol, UK: IOP Publishing), 2815. 012043.

[B17] Li F. S. Fu H. Zhang W. Li Z. F. Xu F. H. (2025). Enhanced black-winged kite algorithm for drone coverage in complex fruit farms. Agriculture 15, 1044. doi: 10.3390/agriculture15101044 30654563

[B84] Li Y. Han L. Liu L. Huang Z. Wang C. He X. (2023). Design and spray performance evaluation of an air–ground cooperation stereoscopic plant protection system for mango orchards. Agronomy 13, 2007. doi: 10.3390/agronomy13082007 30654563

[B3] Li T. Lu H. Luo Q. Li G. Gao M. (2024). The impact of rural population aging on agricultural cropping structure: Evidence from China’s provinces. Agriculture 14, 586. doi: 10.3390/agriculture14040586 30654563

[B65] Li M. Wu F. Wang F. Zou T. Li M. Xiao X. (2024). Cnn-mlp-based configurable robotic arm for smart agriculture. Agriculture 14, 1624. doi: 10.3390/agriculture14091624 30654563

[B89] Li J. Yu D. Ma W. Liu J. J. Liu Y.-J. (2024). Cooperative control of air–ground swarms under dos attacks via cloud–fog computing. IEEE Trans. Network Sci. Eng. 11, 4278–4292. doi: 10.1109/tnse.2024.3409900 25079929

[B50] Liu D. Xiong Z. Liu X. Cai J. Wu D. Wang S. . (2025). “ Simulation study of trajectory tracking of picking robotic arm based on inverse dynamics and fuzzy pid algorithm”, in: Journal of Physics: Conference Series (Bristol, UK: IOP Publishing), 3024. 012041.

[B125] Liu J. Xu C. Jia X. Wu Y. Li T. (2025). Cooperative collision avoidance control with relative velocity information for redundant dual-arm robotic manipulators. J. Bionic Eng. 22, 1111–1125. doi: 10.1007/s42235-025-00671-2 30311153

[B43] Lochan K. Khan A. Elsayed I. Suthar B. Seneviratne L. Hussain I. (2024). Advancements in precision spraying of agricultural robots: A comprehensive review. IEEE Access 12, 129447–129483. doi: 10.1109/access.2024.3450904 25079929

[B92] Ma B. Ji Y. Fang L. (2025). A multi-uav formation obstacle avoidance method combined with improved simulated annealing and an adaptive artificial potential field. Drones 9, 390. doi: 10.3390/drones9060390 30654563

[B25] Maity K. K. Das A. Paul A. Das Mahapatra R. Mahato N. K. (2024). Smart monitoring of vegetable crop by drone: A cyber-physical system model in the arena of accuracy agriculture. 08, 13.

[B48] Malode J. Magadum J. Joshi M. Bhasme S. Iyer S. Shahapur S. (2025). “ Smart agribot: an iot-based precision farming robot with ai-driven irrigation and seed sowing”, in: 2025 11th International Conference on Communication and Signal Processing (ICCSP) (Piscataway, NJ, USA: IEEE), 1368–1373.

[B116] Mammarella M. Comba L. Biglia A. Dabbene F. Gay P. (2022a). Cooperation of unmanned systems for agricultural applications: A theoretical framework. Biosyst. Eng. 223, 61–80. doi: 10.1016/j.biosystemseng.2021.11.008 38826717

[B117] Mammarella M. Comba L. Biglia A. Dabbene F. Gay P. (2022b). Cooperation of unmanned systems for agricultural applications: A case study in a vineyard. Biosyst. Eng. 223, 81–102. doi: 10.1016/j.biosystemseng.2021.12.010 38826717

[B16] Merei A. Mcheick H. Ghaddar A. Rebaine D. (2025). A survey on obstacle detection and avoidance methods for uavs. Drones 9, 203. doi: 10.3390/drones9030203 30654563

[B73] Mishra A. K. Tramacere F. Guarino R. Pugno N. M. Mazzolai B. (2018). A study on plant root apex morphology as a model for soft robots moving in soil. PloS One 13, e0197411. doi: 10.1371/journal.pone.0197411 29874267 PMC5991344

[B102] Mo K. Chu L. Zhang X. Su X. Qian Y. Ou Y. . (2024). Dral: Deep reinforcement adaptive learning for multi-uavs navigation in unknown indoor environment. arXiv preprint arXiv:2409.03930. arXiv:2409.03930. doi: 10.48550/arXiv.2409.03930

[B119] Morchid A. El Alami R. Raezah A. A. Sabbar Y. (2024). Applications of internet of things (iot) and sensors technology to increase food security and agricultural sustainability: Benefits and challenges. Ain Shams Eng. J. 15, 102509. doi: 10.1016/j.asej.2023.102509 38826717

[B91] Mustafa S. A. Kakshar A. S. M. (2025). Analysis and prospect of existing path planning algorithms for multi-drone systems. Qalaai Zanist Sci. J. 10, 1508–1543.

[B70] Nguyen T. H. Muller E. Rubin M. Wang X. Sibona F. McBratney A. . (2025). Towards autonomous in-situ soil sampling and mapping in large-scale agricultural environments. Arxiv Preprint arXiv:2506.05653. doi: 10.48550/arXiv.2506.05653

[B10] Nkwocha C. L. Adewumi A. Folorunsho S. O. Eze C. Jjagwe P. Kemeshi J. . (2025). A comprehensive review of sensing, control, and networking in agricultural robots: from perception to coordination. Robotics. 14, 159. doi: 10.3390/robotics14110159 30654563

[B13] Palta C. (2025). Autonomous drone navigation with dijkstra-based obstacle avoidance in airsim: A comprehensive framework. Int. J. Sci. Innovation Eng. 2, 654–669. doi: 10.70849/IJSCI

[B126] Park Y. Son H. I. (2024). “ Visual scene understanding for efficient cooperative control of agricultural dual-arm robots”, in: 2024 24th International Conference on Control, Automation and Systems (ICCAS) (Piscataway, NJ, USA: IEEE), 1471–1472.

[B45] Peshattiwar A. Wagh S. Meshram S. Nimje A. Patel S. Chakole R. . (2025). “ Smart sprinkler iot system for plants: Real time plant detection & water-nutrition supply management robot using iot & computer vision”, in: 2025 12th International Conference on Emerging Trends in Engineering & Technology-Signal and Information Processing (ICETET-SIP) (Piscataway, NJ, USA: IEEE), 1–6.

[B60] Purwantana B. Hajad M. Ihsanudin M. A. Septanti I. S. Subhan F. Zhang Z. . (2024). Performance of an automatic sorting system based on combination of robotic arm and vision machine for agricultural products. 1–13. doi: 10.2139/ssrn.5253197

[B56] Qian G. (2025). The design and testing of intelligent orchard picking system for agricultural machinery based on image processing technology. Scalable Computing: Pract. Exp. 26, 1489–1495. doi: 10.12694/scpe.v26i3.4324

[B23] Raj A. K. Arulkumar M. Saravanan S. Muthukumar V. (2025). “ Artificial intelligence-based autonomous pesticide spraying drone management for sustainable agriculture”, in: International Conference on Advanced Materials, Manufacturing and Sustainable Development (ICAMMSD 2024) (Dordrecht, The Netherlands: Atlantis Press), 1076–1089.

[B71] Rajanala A. Taylor I. W. McCaskey E. Pierce C. Ligon J. Aydin E. . (2023). The rhizodynamics robot: Automated imaging system for studying long-term dynamic root growth. PloS One 18, e0295823. doi: 10.1371/journal.pone.0295823 38128010 PMC10734993

[B114] Ren Z. Zheng H. Chen J. Chen T. Xie P. Xu Y. . (2024). Integrating uav, ugv and uav-ugv collaboration in future industrialized agriculture: Analysis, opportunities and challenges. Comput. Electron. Agric. 227, 109631. doi: 10.1016/j.compag.2024.109631 38826717

[B97] Rizaldi A. Kim Y. (2025). Optimization of dual-type multi-drone formations for load carrying missions. Int. J. Control Autom. Syst. 23, 498–509. doi: 10.1007/s12555-024-0544-6 30311153

[B83] Rodrigues Barbosa Júnior M. Garcia dos Santos R. de Azevedo Sales L. da Silva Martins J. V. de Almeida Santos J. G. Pereira de Oliveira L. (2025). Designing and implementing a ground-based robotic system to support spraying drone operations: A step toward collaborative robotics. Actuators, 14 (8), 365.

[B99] Rubinacci R. Nazzari A. Lovera M. (2025). Anytime trajectory optimization for multi-drone systems with guaranteed collision avoidance. IEEE Control Syst. Lett. 9, 1255–1260. doi: 10.1109/lcsys.2025.3580039 25079929

[B55] Sahu U. K. KS M. K A. P M. M. Jaiswal A. Yadav U. K. . (2025). Autonomous object tracking with vision based control using a 2dof robotic arm. Sci. Rep. 15, 13404. doi: 10.1038/s41598-025-97930-3 40251241 PMC12008391

[B120] Sarpal D. Sinha R. Jha M. Tn P. (2022). Agriwealth: Iot based farming system. Microprocess. Microsyst. 89, 104447. doi: 10.1016/j.micpro.2022.104447 38826717

[B8] Singh S. Vaishnav R. Gautam S. Banerjee S. (2024). “ Agricultural robotics: A comprehensive review of applications, challenges and future prospects”, in: Proceedings of the 2024 2nd international conference on artificial intelligence and machine learning applications theme: healthcare and internet of things (AIMLA) (Piscataway, NJ, USA: IEEE), 1–8.

[B27] Sitompul E. Gubarda M. R. Sihombing P. Simarmata T. Turnip A. (2024). “ Sprayer system on autonomous farming drone based on decision tree”, in: Proceedings of the 2024 IEEE International Conference on Artificial Intelligence and Mechatronics Systems (AIMS) (Piscataway, NJ, USA: IEEE), 1–6.

[B18] Siwek M. Baranowski L. Ładyzyńska-Kozdraś E. (2024). The application and optimisation of a neural network pid controller for trajectory tracking using uavs. Sensors 24, 8072. doi: 10.3390/s24248072 39771807 PMC11679864

[B72] Song Z. Zhao T. Jin J. (2023). Early identification of root damages caused by western corn rootworms using a minimally invasive root phenotyping robot—misiroot. Sensors 23, 5995. doi: 10.3390/s23135995 37447843 PMC10346699

[B9] Spagnuolo M. Todde G. Caria M. Furnitto N. Schillaci G. Failla S. (2025). Agricultural robotics: A technical review addressing challenges in sustainable crop production. Robotics 14, 9. doi: 10.3390/robotics14020009 30654563

[B31] Srinil P. Thongnim P. (2024). Deep learning enhanced hand gesture recognition for efficient drone use in agriculture. Int. J. Advanced Comput. Sci. Appl. 15, 1257–1264. doi: 10.14569/ijacsa.2024.01505127

[B57] Sroymuk P. Juntarakod P. Sukontanakarn V. (2025). Integration of image processing with 6-degrees-of-freedom robotic arm for advanced automation. TELKOMNIKA (Telecommunication Computing Electron. Control) 23, 758–769. doi: 10.12928/telkomnika.v23i3.26617

[B49] Subbulakshmi A. Sandhiya M. Palanivel S. Narayanan L. K . (2025). “ Fpga-based automatic plant watering system: A real-time implementation and verification using xilinx”, in: 2025 Fourth International Conference on Smart Technologies, Communication and Robotics (STCR) (Piscataway, NJ, USA: IEEE), 1–6.

[B61] Subrata I. D. M. Baiquni A. D. (2024). Application of stereo vision to control the movement of the robot arm towards the position of red chilies. Jurnal Teknik Pertanian Lampung (Journal Agric. Engineering) 13, 615–627. doi: 10.23960/jtep-l.v13i3.615-627

[B82] Sun Y. Guo X. Song J. Zhou S. Jiang Z. Liu X. . (2019). Adaptive learning-based task offloading for vehicular edge computing systems. IEEE Trans. Veh. Technol. 68, 3061–3074. doi: 10.1109/tvt.2019.2895593 25079929

[B81] Sun F. Lin B. Zhang H. Li T. Li M. Shi M. . (2025). “ Air-ground cooperation optimal coverage path planning with connectivity maintenance”, in: 2025 Joint International Conference on Automation-Intelligence-Safety (ICAIS) & International Symposium on Autonomous Systems (ISAS) (Piscataway, NJ, USA: IEEE), 1–6.

[B51] Taddei Dalla Torre F. Faris O. Johnson P. H. Calisti M. (2025). “ Toward autonomous 909 blackberry harvesting with a soft gripper and vision-controlled robotic arm”, in: 2025 IEEE 8th International Conference on Soft Robotics (RoboSoft) (Piscataway, NJ, USA: IEEE), 1–8.

[B94] Tang R. Tang J. Abu Talip M. S. Aridas N. K. Xu X. (2025). Enhanced multi agent coordination algorithm for drone swarm patrolling in durian orchards. Sci. Rep. 15, 9139. doi: 10.1038/s41598-025-88145-7 40097450 PMC11914038

[B86] Tao H. Zhao W. Zhao L. Wang J. (2025). Research on disaster environment map fusion construction and reinforcement learning navigation technology based on air–ground collaborative multi-heterogeneous robot systems. Sensors 25, 4988. doi: 10.3390/s25164988 40871852 PMC12390346

[B7] Thaker R. K. (2023). Advances in agricultural robotics: Present applications, challenges, and future prospects. IJLRP-International J. Leading Res. Publ. 4, 1–8. doi: 10.5281/zenodo.14001929

[B2] Tong T. Ye F. Zhang Q. Liao W. Ding Y. Liu Y. . (2024). The impact of labor force aging on agricultural total factor productivity of farmers in China: implications for food sustainability. Front. Sustain. Food Syst. 8, 1434604. doi: 10.3389/fsufs.2024.1434604

[B1] FAO IFAD UNICEF WFP WHO . (2024). The state of food security and nutrition in the world 2024. 286.

[B12] Verma S. K. (2025). Exploring the role of ai in autonomous drone technology for precision pest control in agriculture. 13 (1), 19–28.

[B75] Wang X. Cao Z. (2025). The synergy of agricultural open space and drone based on color difference and gradient characteristics and sensor integration. Int. J. High Speed Electron. Syst. 35, 2540804. doi: 10.1142/s0129156425408046 31116912

[B20] Wang P. Hanif A. S. Yu S.-H. Lee C.-G. Kang Y. H. Lee D.-H. . (2024). Development of an autonomous drone spraying control system based on the coefficient of variation of spray distribution. Comput. Electron. Agric. 227, 109529. doi: 10.1016/j.compag.2024.109529 38826717

[B90] Wang L. Huang W. Li H. Li W. Chen J. Wu W. (2024). A review of collaborative trajectory planning for multiple unmanned aerial vehicles. Processes 12, 1272. doi: 10.3390/pr12061272 30654563

[B105] Wang N. Yang X. Wang T. Xiao J. Zhang M. Wang H. . (2023). Collaborative path planning and task allocation for multiple agricultural machines. Comput. Electron. Agric. 213, 108218. doi: 10.1016/j.compag.2023.108218 38826717

[B14] Wei P. Ragbir P. Vougioukas S. G. Kong Z. (2025). Vision-based navigation of unmanned aerial vehicles in orchards: An imitation learning approach. Comput. Electron. Agric. 238, 110802. doi: 10.1016/j.compag.2025.110802 38826717

[B35] Wen J. Yao L. Zhou J. Yang Z. Xu L. Yao L. (2025). Path tracking control of agricultural automatic navigation vehicles based on an improved sparrow search-pure pursuit algorithm. Agriculture 15, 1215. doi: 10.3390/agriculture15111215 30654563

[B74] Wu J. Wu Q. Pagès L. Yuan Y. Zhang X. Du M. . (2018). Rhizochamber-monitor: a robotic platform and software enabling characterization of root growth. Plant Methods 14, 44. doi: 10.1186/s13007-018-0316-5 29930694 PMC5991437

[B80] Wu D. Zhang J. Abusorrah A. Zhou M. (2025). A bin-packing-based method for path planning of air–ground cooperative system. IEEE Trans. Veh. Technol. 74, 279–291. doi: 10.1109/tvt.2024.3454101 25079929

[B52] Xu H. Xin Y. Wang Z. Chen S. Li B. (2025). The design and finite element analysis of the mushroom picking flexible robotic arm. J. Vibroeng. 27, 550–566. doi: 10.21595/jve.2025.24330

[B100] Xu G. Zhao J. Wang X. (2024). Wireless sensing charging based on multi-drone cooperation. IET Networks 13, 199–207. doi: 10.1049/ntw2.12107

[B40] Xu W. Zhu Y. Xiao M. Liu M. Yang Y. Yue X. . (2025). A multi-agent-based cooperative optimization control method for the motor energy consumption and tracking accuracy of an unmanned electric tractor. Eng. Appl. Artif. Intell. 159, 111665. doi: 10.1016/j.engappai.2025.111665 38826717

[B127] Yan B. Li X. (2025). An intelligent operation area allocation and automatic sequential grasping algorithm for dual-arm horticultural smart harvesting robot. Horticulturae 11, 740. doi: 10.3390/horticulturae11070740 30654563

[B37] Yongtao W. Fanxi K. Xing Z. Zhiwei K. (2025). “ Research on path planning for power inspection robots based on improved adaptive multi-modal ant colony optimization algorithm”, in: 2025 IEEE 12th Joint International Information Technology and Artificial Intelligence Conference (ITAIC) (Piscataway, NJ, USA: IEEE), 12, 668–673.

[B107] Yu Y. Liu Y. Wang J. Noguchi N. He Y. (2023). Obstacle avoidance method based on double DQN for agricultural robots. Comput. Electron. Agric. 204, 107546. doi: 10.1016/j.compag.2022.107546 38826717

[B54] Yu Z. Lu C. Zhang Y. Jing L. (2024). Gesture-controlled robotic arm for agricultural harvesting using a data glove with bending sensor and optitrack systems. Micromachines 15, 918. doi: 10.3390/mi15070918 39064429 PMC11278971

[B11] Yumnam C. Marak A. B. Kumar M. Khatri S. Kumar S. Soni S. . (2025). Utilising drones in agriculture: A review on remote sensors, image processing and their application. Mod. Agric. 3, e70021. doi: 10.1002/moda.70021 41531421

[B87] Zhang J. Chen H. Dai F. Wang Y. Li H. Zhang Y. (2025). A fast multi-uav assistance positioning architecture for large-scale farming operations. IEEE Internet Things J. 12, 37645–37658. doi: 10.1109/jiot.2025.3584091 25079929

[B131] Zhang P. Dai N. Wang Z. Yuan J. Xin Z. Liu X. . (2025). A parallel dual-arm robotic control method of white asparagus based on moving-looking-harvesting coordination and asynchronous harvest cooperation. Comput. Electron. Agric. 232, 110046. doi: 10.1016/j.compag.2025.110046 38826717

[B38] Zhang X. Duan Y. Li X. Yang J. Li D. Zhao R. . (2025). Path planning for greenhouse robots using a hybrid dung beetle algorithm. Intell. Serv. Rob. 18, 1–25. doi: 10.1007/s11370-025-00592-3 30311153

[B110] Zhang T. Jiao X. Lin Z. (2022). Finite time trajectory tracking control of autonomous agricultural tractor integrated nonsingular fast terminal sliding mode and disturbance observer. Biosyst. Eng. 219, 153–164. doi: 10.1016/j.biosystemseng.2022.04.020 38826717

[B79] Zhang N. Zhang B. Zhang Q. Gao C. Feng J. Yue L. (2025). Large-area coverage path planning method based on vehicle–uav collaboration. Appl. Sci. 15, 1247. doi: 10.3390/app15031247 30654563

[B39] Zhang L. Zhang R. Zhang D. Yi T. Ding C. Chen L. (2025). Path curvature incorporated reinforcement learning method for accurate path tracking of agricultural vehicles. Comput. Electron. Agric. 234, 110243. doi: 10.1016/j.compag.2025.110243 38826717

[B130] Zhu Y. Lu W. Ren G. Ying Y. Vougioukas S. Peng C. (2025). Optimal scheduling of a dual-arm robot for efficient strawberry harvesting in plant factories. arXiv preprint arXiv:2507.04240. doi: 10.48550/arXiv.2507.04240

[B36] Zhu X. Zhao J. Ni H. (2025). Dynamic path planning of mobile robots based on improved gwo algorithm. J. Ambient Intell. Hum. Comput. 16, 831–840. doi: 10.1007/s12652-025-04980-6 30311153

